# Circular RNA FEACR inhibits ferroptosis and alleviates myocardial ischemia/reperfusion injury by interacting with NAMPT

**DOI:** 10.1186/s12929-023-00927-1

**Published:** 2023-06-27

**Authors:** Jie Ju, Xin-Min Li, Xue-Mei Zhao, Fu-Hai Li, Shao-Cong Wang, Kai Wang, Rui-Feng Li, Lu-Yu Zhou, Lin Liang, Yin Wang, Yu-Hui Zhang, Kun Wang

**Affiliations:** 1 Key Laboratory of Birth Regulation and Control Technology of National Health Commission of China, Shandong Provincial Maternal and Child Health Care Hospital Affiliated to Qingdao University, Jinan, 250014 China; 2 Institute for Translational Medicine, The Affiliated Hospital of Qingdao University, College of Medicine, Qingdao University, Qingdao, 266021 China; 3 State Key Laboratory of Cardiovascular Disease, Heart Failure Center, Fuwai Hospital, National Center for Cardiovascular Diseases, Chinese Academy of Medical Sciences, Peking Union Medical College, Beijing, 100037 China; 4 Department of Cardiology, The Affiliated Hospital of Qingdao University, Qingdao, 266021 China

**Keywords:** circRNA, FEACR, Cardiomyocyte ferroptosis, NAMPT

## Abstract

**Background:**

Emerging research has reported that circular RNAs (circRNAs) play important roles in cardiac cell death after myocardial ischemia and reperfusion (I/R). Ferroptosis, a new form of cell death discovered in recent years, has been proven to participate in the regulation of myocardial I/R. This study used circRNA sequencing to explore the key circRNA in the regulation of cardiac ferroptosis after I/R and study the mechanisms of potential circRNA function.

**Methods:**

We performed circRNA sequencing to explore circRNAs differentially expressed after myocardial I/R. We used quantitative polymerase chain reactions to determine the circRNA expression in different tissues and detect the circRNA subcellular localization in the cardiomyocyte. Gain- and loss-of-function experiments were aimed to examine the function of circRNAs in cardiomyocyte ferroptosis and cardiac tissue damage after myocardial I/R. RNA pull-down was applied to explore proteins interacting with circRNA.

**Results:**

Here, we identified a ferroptosis-associated circRNA (FEACR) that has an underlying regulatory role in cardiomyocyte ferroptosis. FEACR overexpression suppressed I/R-induced myocardial infarction and ameliorated cardiac function. FEACR inhibition induces ferroptosis in cardiomyocytes and FEACR overexpression inhibits hypoxia and reoxygenation-induced ferroptosis. Mechanistically, FEACR directly bound to nicotinamide phosphoribosyltransferase (NAMPT) and enhanced the protein stability of NAMPT, which increased NAMPT-dependent Sirtuin1 (Sirt1) expression, which promoted the transcriptional activity of forkhead box protein O1 (FOXO1) by reducing FOXO1 acetylation levels. FOXO1 further upregulated the transcription of ferritin heavy chain 1 (*Fth1*), a ferroptosis suppressor, which resulted in the inhibition of cardiomyocyte ferroptosis.

**Conclusions:**

Our finding reveals that the circRNA FEACR-mediated NAMPT-Sirt1-FOXO1-FTH1 signaling axis participates in the regulation of cardiomyocyte ferroptosis and protects the heart function against I/R injury. Thus, FEACR and its downstream factors could be novel targets for alleviating ferroptosis-related myocardial injury in ischemic heart diseases.

**Supplementary Information:**

The online version contains supplementary material available at 10.1186/s12929-023-00927-1.

## Introduction

Heart disease is a major killer that is seriously harmful to health, according to a report from the American Heart Association [1]. Although various approaches and strategies have been developed, cardiac disease still has a high mortality. Rapid restoration of blood flow (reperfusion) is a major treatment strategy for the heart and improves prognosis and survival. Although reperfusion is necessary for oxygen and nutrient supply, reperfusion after a period of ischemia can lead to cardiac dysfunction and even cardiac failure. Cardiac ischemia/reperfusion (I/R) leads to the acute loss of cardiomyocytes caused by irreversible injury and cell death, which is an important cause of various cardiac pathologies, ventricular remodeling and heart failure. After I/R, a large proportion of cardiomyocytes undergo apoptotic and nonapoptotic cell death, such as necroptosis, ferroptosis and pyroptosis [2–4]. Therefore, attenuating the death of cardiomyocytes after I/R is an important strategy to reduce the severity of myocardial infarction and the risk of cardiac injury.

Circular RNAs (circRNAs), which are a type of noncoding RNA, were first discovered in plant viruses in 1976 [5]. CircRNA has a closed circular structure, is more stable and can avoid degradation, which suggests better stability than linear RNA. Studies have shown that circRNAs play important biological roles, such as miRNA sponges, RNA binding protein sponges and mediating the translation of short peptides, mainly through their sequence characteristics [6, 7]. The circRNA CNEACR inhibits cardiomyocyte necrosis induced by hypoxia/reoxygenation (H/R) and attenuates myocardial necrosis in I/R-induced mouse hearts. CNEACR binds to histone deacetylase 7 (HDAC7) in the cytoplasm and restricts its nuclear entry. This inhibits forkhead box protein A2 (Foxa2) transcription, which represses the receptor-interacting protein kinase 3 (Ripk3) gene by binding to its promoter region [6]. Circ-NNT is upregulated in myocardial infarction patients, as well as in cultured cardiomyocytes after H/R and cardiac tissues after I/R. CircRNA circ-NNT mediates myocardial ischemia/reperfusion injury by activating pyroptosis by sponging miR-33a-5p and regulating USP46 expression [8]. To date, a variety of studies have revealed that circRNAs play important roles in many cellular processes. However, the functions of circRNAs in I/R-induced myocardial ferroptosis are still unclear and require further study.

Our present study revealed that the circRNA FEACR (ferroptosis-related circRNA) is involved in the regulation of cardiomyocyte ferroptosis and improves myocardial I/R injury. We found that FEACR directly interacts with nicotinamide phosphoribosyl transferase (NAMPT) and maintains its stability, thus affecting NAD-dependent deacetylase sirtuin1 (Sirt1)-mediated forkhead box protein O1 (FOXO1) deacetylation to regulate ferritin heavy chain 1 (*Fth1*) transcription. Our findings reveal a novel regulatory role of circRNAs in ferroptosis and identify a new molecular mechanism of circRNAs in the treatment of heart diseases.

## Methods

### Culture of cardiomyocytes

Newborn mice aged 1–3 days were used to isolate primary cardiomyocytes. First, we executed the mice, harvested hearts, and collected them in a 6-well plate. All the isolated hearts were washed with phosphate buffer saline (PBS) three times and cut into tissue pieces of 1mm^3^. The heart tissues were dissociated in 5 mL PBS with 0.14 mg/mL collagenase and 1.2 mg/mL pancreatin at 37 °C and repeated several times until all the heart tissues disappeared. The lysates were collected in a 50 mL centrifuge tube containing 5 mL horse serum and then centrifuged at 1000 rpm for 5 min. Dulbecco’s modified Eagle medium/F-12 (Servicebio) supplemented with 5% fetal bovine serum was used to resuspend the pellet. The cell suspension was filtered with a 100 mesh filter in a 10 cm dish. The cells were adherent in an incubator for 1.5 h and suspending cells were primary cardiomyocytes. The cardiomyocytes were cultured in a 5% DMEM/F12 medium with 0.1 mM bromodeoxyuridine in an atmosphere of 5% CO_2_ at 37 °C.

### Hypoxia/reoxygenation (H/R) treatment

H/R was performed in cardiomyocytes as previously describe [9, 10]. In brief, cardiomyocytes were switched to a glucose-free medium and kept in a hypoxia chamber (containing 5% CO_2_ and 95% N_2_) at 37 °C for 18 h, Subsequently, the cells were cultured in 5% medium and placed in an incubator (95% air and 5% CO_2_) at 37 °C for 6 h to establish in vitro H/R model.

### Animal experiments

We purchased the adult male C57BL/6J mice (8–10 weeks old) from Pengyue Experimental Animal Breeding Co., LTD (Jinan, China). The mice used for the experiment were of the same age and body weight and were assigned randomly to each group. All animal researches conform to the Directive 2010/63/EU of the European Parliament and have been approved by the Biomedical Research Ethics Committee of Qingdao University (License No: 20200610C576620221208124).

The ischemia and reperfusion (I/R) model is conducted by ligation of the left anterior descending coronary artery (LAD) for 45 min and reperfusion for 3 h, as previously described [11]. In the sham group, a similar procedure was done except for the ligation of LAD. Adenovirus adv-FEACR, adv-NAMPT, adv-FTH1, and their negative control (NC) were purchased from HanBio biotechnology. For overexpression of FEACR, NAMPT, and FTH1, the mice were treated with adv-FEACR, adv-NAMPT, and adv-FTH1 (2 × 10^10^ moi) by tail vein injection. For knockdown of FEACR, NAMPT, and FTH1, the mice were treated with adv-shFEACR, adv-shNAMPT, and adv-shFTH1 (2 × 10^10^ moi) by tail vein injection. Three days after injection, mice were treated with I/R, and heart samples were collected to conduct molecular and morphological studies. We conducted the experiments in accordance with the guidelines of Qingdao University’s Animal Care Committee.

### Western blot

RIPA buffer containing 100 μM PMSF (Solarbio) is used to lyse cardiomyocytes and cardiac tissues. Cardiomyocytes or cardiac tissues were suspended in RIPA buffer and were lysed on ice for 20 min. The lysate was centrifuged at 4 °C, 13,000 rpm for 20 min, and the concentration of total protein was measured by BCA kit (Vazyme). SDS-PAGE electrophoresis was used to separate cell lysates, which were then transferred onto PVDF membranes. The primary antibody was incubated with the membrane at 4 °C overnight, the blot was incubated with HRP-conjugated secondary antibodies for 1 h at room temperature followed by development with ECL Western blot substrate. We use primary antibodies in the following concentrations: GPX4 (ZEN-BIOSCIENCE, 381958) 1:3000, SLC7A11 (ZEN-BIOSCIENCE, 382036) 1:3000, FTH1 (ZEN-BIOSCIENCE, 381204) 1:2000, NAMPT (Klean AB, P100462) 1:3000, Sirt1 (Zenbio, R25721) 1:2000, FOXO1 (Zenbio, 380978) 1:1000, Ac-FOXO (Affinity, AF2305) 1:1000, RIPK1(Affinity, AF7877) 1:2000, RIPK3 (Affinity, DF10141) 1:2000, MLKL (Affinity, DF7412) 1:2000, BAX (Klean AB, P100045) 1:1000 and GAPDH (Abclonal, A19056) 1:5000, overnight at 4 °C.

### RNA pull-down assay and MS analysis

A FEACR labeled with 5′-biotin and negative control (NC) were obtained from GenePharma, the sequence as follows: Biotin-FEACR: 5′biotin-AAUGCCUGCCAUGCUGAGUAAUGGG; Scramble Biotin-FEACR: 5′biotin-UAAUGCACGAUGUGGUCGUAACGGC. The cardiomyocytes were washed with PBS several times and then collected in a 1.5 mL EP tube. The cell pellet was resuspended in lysis buffer (20 mM Tris–HCl, 200 mM NaCl, 2.5 mM MgCl_2_, 1 mM DTT, 1× protease inhibitors) and digested for 30 min. The lysates were centrifuged at 12,000 rpm, 4 °C for 20 min, and aliquoted 50 μL lysates were used as input. The remaining lysates were incubated respectively with biotin-FEACR and biotin-NC and formed a mixture overnight. A solution of streptavidin-magnetic beads (BioMag) was then incubated with mixture at 4 °C for 3 h and washed five times with lysis buffer. Then the beads were boiled with 2× SDS loading buffer (SparkJade) for 5 min at 95 °C. The PAGE gel was stained with coomassie bright blue until the band is clear and extracted gel lines underwent LC–MS/MS analysis at a commercial laboratory. The gel band was digested by trypsin and HPLC liquid system Easy nLC was used to separate each sample at a nanoliter flow rate. After chromatographic separation, mass spectrometry was applied to detect the samples by using a Q-Exactive mass spectrometer. The original data of mass spectrometry analysis were RAW files, and MaxQuant software (version No. 1.5.3.17) was used for database identification and quantitative analysis (Shanghai Applied Protein Technology). The raw MS data was uploaded to the iProX database, and the subproject ID is IPX0005237002.

### Statistical analysis

The results are represented as the mean ± s.d of at least three independent experiments. The data were evaluated with a two-sided student’s t-test or one-way analysis of variance (one-way ANOVA) for multiple comparisons. Statistical analyses were performed using GraphPad Prism software (version 7.0, San Diego, CA), and P-value < 0.05 were considered significant.

## Results

### Identification and characterization of cardiac ferroptosis-associated circRNAs

**Fig. 1:**
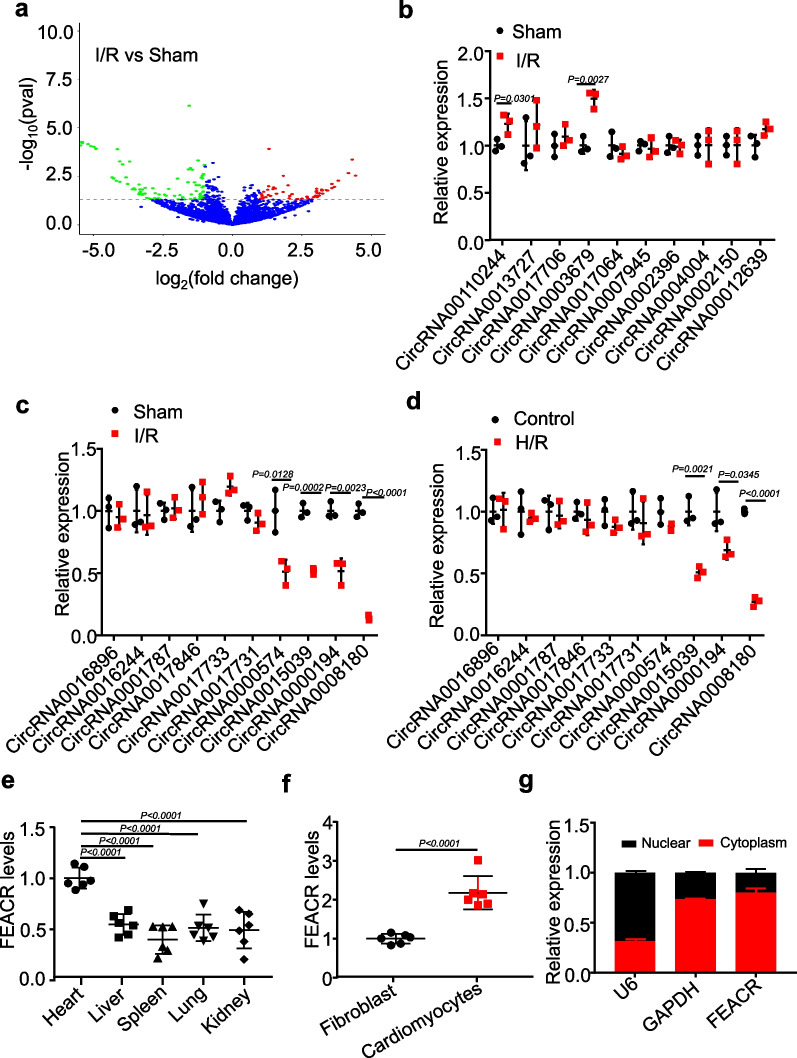
Identification and characterization of cardiac ferroptosis-associated circRNAs. **a** The relative circRNA expression from Sham vs. I/R-treated mice was exhibited by volcano plot. The circRNA in the upper left quadrant and upper right quadrant are significantly differentially expressed. **b** The expression levels of upregulated circRNAs (screened from RNA-seq data) in heart tissues with I/R injury were determined by qPCR. **c**, **d** The expression levels of downregulated circRNAs were determined by qPCR in heart tissues with I/R injury or in cardiomyocytes treated with H/R. **e** The expression level of FEACR in different tissues of mice. **f** The expression level of FEACR was determined by qPCR in cardiomyocytes and fibroblasts isolated from mice. **g** The level of FEACR in the cellular component of the nucleus and cytoplasm was detected by qPCR. The expression of U6 and GAPDH was used to determine the purity of cytoplasmic or nuclear fractions. **b**, **c** n = 3 mice per group; **d** n = 3 biological replicates; **e**, **f** n = 6 biological replicates; **g** n = 3 biological replicates. Data were mean ± SD, and the P-value was calculated by Student’s t-test (**b**–**d**, **f**) or One-way ANOVA (**e**). The experiment technically repeats three times

The death of terminally differentiated cardiomyocytes is a crucial pathogenic factor in the development of heart injury. Ferroptosis has been proven to be an important contributor to some cardiac problems, including I/R injury [3, 12, 13]. To explore the conditions under which different ischemic times lead to ferroptosis in myocardial tissue, we examined the changes in ferroptosis biomarkers after different ischemic times, as shown in Additional file 1: Fig. S1a, b. To comprehensively explore the potential functions of circRNAs associated with ferroptosis in cardiomyocytes, we performed RNA-seq analysis of circRNAs in mouse hearts after I/R injury (Fig. 1a, Additional file 2: Table S1). Among the differentially expressed circRNAs, we selected the top 10 dysregulated circRNAs (10 upregulated and 10 downregulated, ≥ twofold up- and downregulated; *P* < 0.05) and confirmed their expression levels by RT‒qPCR. The results showed that the expression of the top ten upregulated RNAs was not significantly altered by I/R or H/R (Fig. 1b, Additional file 1: Fig. S1c). Among the top ten downregulated circRNAs, the expression of circRNA0008180 was most markedly reduced in I/R-injured mouse hearts compared to sham hearts (Fig. 1c). We also found that the expression of circRNA0008180 was significantly decreased in H/R-treated cardiomyocytes (Fig. 1d). This indicates that circRNA0008180 might participate in the regulation of cardiac I/R injury and is worthy of further investigation. We thus named this circRNA0008180 as ferroptosis-associated circRNA (FEACR) for further study.

FEACR is located on chr19:45,619,304–45,628,960 (mouse mm39) and is derived from the back-splicing of linear FBXW4 mRNA exons 2–5. We experimentally verified the ring structure of FEACR in mouse hearts using divergent primers and RT‒PCR (Additional file 1: Fig. S1d). We then detected the expression levels of FEACR in different tissues and found that it was also expressed in other organs, but the expression levels of FEACR were highest in the heart (Fig. 1e). In addition, the expression of FEACR was markedly higher in neonatal mouse cardiomyocytes than in fibroblasts (Fig. 1f). The expression of FEACR in cardiomyocytes was higher than that in fibroblasts isolated from adult mice, and FEACR was also downregulated in cardiomyocytes isolated from I/R-induced mice compared with sham mice (Additional file 1: Fig. S1e). In addition, FEACR was primarily expressed in the cytoplasm, and a small amount was expressed in the nucleus (Fig. 1g). We also confirmed the cellular distribution of FEACR by FISH and found that FEACR was mainly located in the cytoplasm (Additional file 1: Fig. S1f). These data indicate that FEACR is downregulated by cardiac I/R injury and might play important roles in cardiomyocyte death.

### FEACR protects cardiomyocytes from I/R-induced ferroptosis

**Fig. 2:**
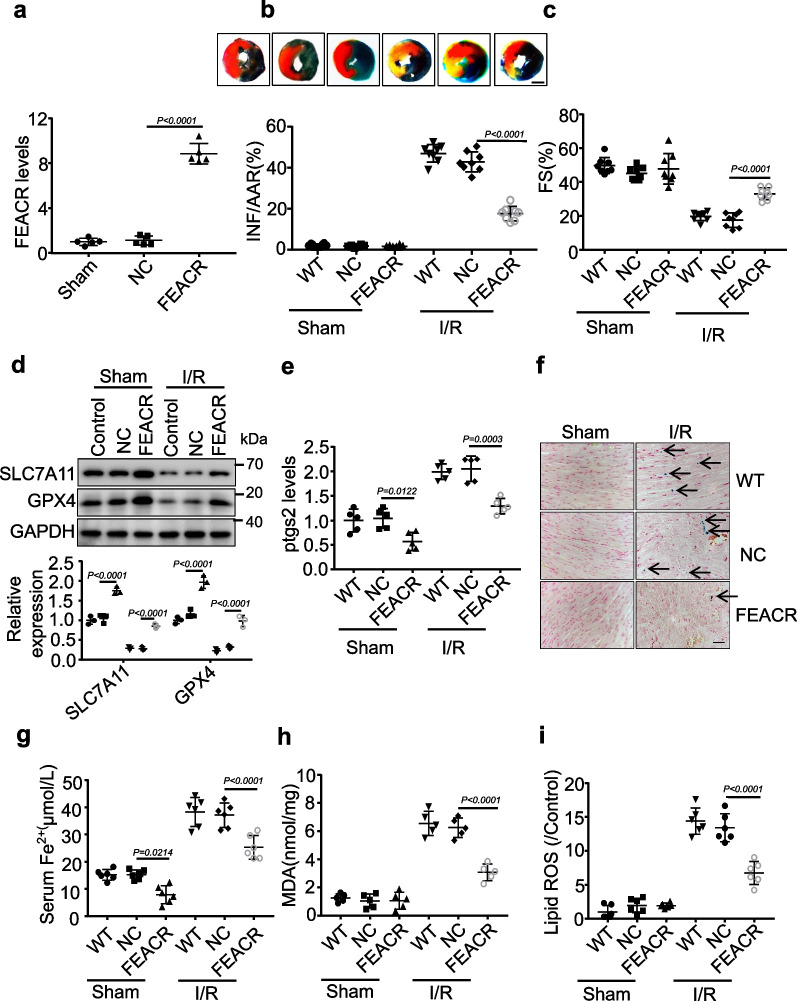
FEACR attenuates ferroptosis in vivo. **a** The adenovirus expressing FEACR was delivered into the mice and the expression level of FEACR was measured by qPCR in vivo. **b**–**i** FEACR or negative control (NC) was overexpressed by delivery of the adenovirus expressing FEACR or NC and then performed I/R injury or sham in mice. **b** The midventricular heart section was stained by Evans blue-TTC and representative images are shown in upper panels as blue-healthy area, red-risk area, white-infarcted area, scale bar, 2 mm. The chart below displays infarct sizes. AAR refers to the area at risk, and INF refers to the infarct area. **c** The left ventricular fractional shortening (FS) was monitored by the transthoracic ultrasonic imaging system. **d** Ferroptosis-related proteins were examined by Western blot (upper) and were quantified (lower). **e** Ptgs2 level was detected by qPCR. **f** Representative images of Prussian blue iron staining in cardiac tissue. The black arrow indicated ferric iron. **g** The level of ferrous iron content in the serum. **h** The level of lipid peroxide MDA in each group. **i** Quantitative statistics of ROS content stained with C11-BODIPY in cardiomyocytes. **a** n = 5 mice per group; **b**, **c** n = 8 mice per group; **d** n = 3 biological replicates; **e**, **h** n = 5 mice per group; **g**, **i** n = 6 mice per group. Data were mean ± SD, and the P*-*value was calculated by One-way ANOVA. The experiment technically repeats three times

To explore the functional role of FEACR in ferroptosis during I/R injury, we first injected adenovirus expressing FEACR into wild type (WT) mice and confirmed the delivery efficiency (Fig. 2a). Evans blue-TTC staining revealed that overexpression of FEACR suppressed the I/R-induced increase in infarct size compared to WT mouse hearts subjected to I/R injury (Fig. 2b), although the groups had comparable areas at risk (Additional file 1: Fig. S2a). Left ventricular fractional shortening, which is an important indicator of left ventricular systolic function, was improved in I/R-injured mouse hearts after FEACR overexpression (Fig. 2c). In addition, other cardiac function indicators were improved after FEACR overexpression in I/R hearts (Additional file 1: Fig. S2b–f). SLC7A11 can transport cysteines into the cell for GSH synthesis, protect cells from oxidative stress damage and maintain redox balance, thus preventing ferroptosis caused by lipid peroxidation [14]. GPX4 can remove membrane lipid hydrogen peroxide products and prevent intracellular peroxide accumulation to inhibit ferroptosis [15]. The downregulation of GPX4 and SLC7A11 is used as a marker of ferroptosis [16]. We then found that the levels of SLC7A11 and GPX4 were downregulated after I/R injury and that FEACR attenuated the I/R-induced downregulation of SLC7A11 and GPX4 (Fig. 2d). *Ptgs2*, also known as cyclooxygenase 2, is upregulated by the ferroptosis inducer erastin or RSL3 treatment and is a molecular marker of ferroptosis [17]. FEACR overexpression reduced I/R-induced upregulation of *Ptgs2* mRNA (Fig. 2e) and PTGS2-positive immunofluorescence intensity (Additional file 1: Fig. S3a). Prussian blue staining of iron (Fig. 2f) and the iron content in serum (Fig. 2g) were both attenuated in FEACR-overexpressing mice after I/R injury. Furthermore, we detected cardiac malondialdehyde (MDA) levels and found that FEACR overexpression inhibited the I/R-induced increase in MDA (Fig. 2h). Ferroptosis occurs when the antioxidant capacity of cells decreases and is insufficient to remove the excessive accumulation of lipid ROS. We found that FEACR attenuated lipid ROS production after I/R (Fig. 2i). We next extended the I/R time and found that FEACR also attenuated the increases in *Ptgs2* and MDA at later time points after I/R injury (Additional file 1: Fig. S3b, c). Necrosis and apoptosis of cardiomyocytes play important roles in myocardial I/R injury. Therefore, we tested the effects of FEACR on I/R-induced apoptosis and necrosis. We observed that FEACR had no effect on the expression of RIPK1, RIPK3 and MLKL after I/R, which indicates that FEACR does not influence necrosis (Additional file 1: Fig. S4a, b). Furthermore, FEACR did not change the expression of apoptosis biomarkers BAX (Additional file 1: Fig. S4c, d) or the ratio of TUNEL-positive cardiomyocytes (Additional file 1: Fig. S4e, f) after I/R injury, which suggests that FEACR is not involved in the regulation of apoptosis. Taken together, these results suggest that FEACR attenuates I/R-induced ferroptosis and myocardial dysfunction in vivo.

### FEACR inhibits H/R-induced ferroptosis in vitro

**Fig. 3:**
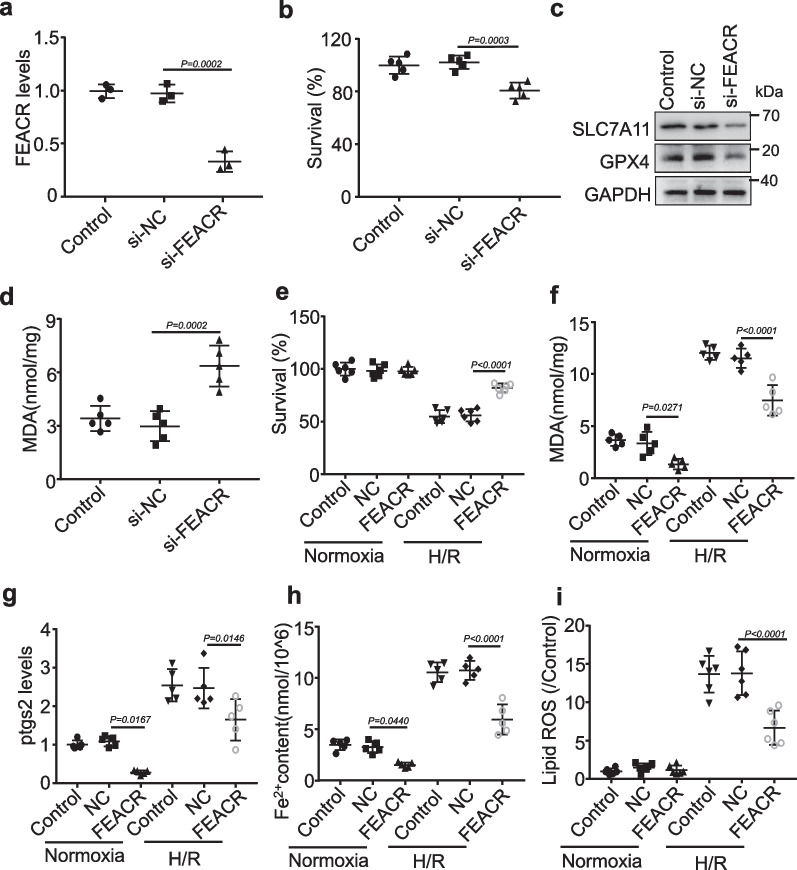
FEACR inhibits H/R-induced ferroptosis in cardiomyocytes. **a** The expression of FEACR was inhibited in cardiomyocytes measured by qPCR. **b** CCK-8 kit determines the cell survival rate after FEACR inhibition. **c** The expression of ferroptosis-related proteins after the knockdown of FEACR was revealed by Western blot. **d** The content of lipid peroxidation production MDA in cardiomyocytes upon FEACR inhibition. **e**, **f** Cell survival rate and MDA content were measured after overexpression of FEACR in cardiomyocytes subjected to H/R treatment. **g** QPCR analysis measured the ptgs2 level after overexpression of FEACR in H/R-treated cardiomyocytes. **h** Iron content was determined by ferrous iron kit in cardiomyocytes. **i** Quantitative statistics of ROS content stained with C11-BODIPY in cardiomyocytes. **a** n = 3 biological replicates; **b** n = 5 biological replicates; **c** n = 3 biological replicates; **d**, **f**–**h** n = 5 biological replicates; **e** n = 6 biological replicates; **i** n = 6 biological replicates. Data were mean ± SD, and the P*-*value was calculated by One-way ANOVA. The experiment technically repeats three times

Next, we examined the roles of FEACR in the regulation of ferroptosis in cardiomyocytes. To further verify the function of FEACR in protecting cardiomyocytes against ferroptosis, we established H/R-induced ferroptosis to mimic I/R injury in cardiomyocytes [9]. Pretreatment with the ferroptosis inhibitor ferrostatin-1 (Fer-1) inhibited H/R-induced decreases in cell survival (Additional file 1: Fig. S5a), increases in MDA content (Additional file 1: Fig. S5b), and SLC7A11 and GPX4 downregulation (Additional file 1: Fig. S5c), which indicates that ferroptosis is involved in H/R-induced cell death. We then transfected FEACR siRNA into cardiomyocytes and examined the knockdown efficiency by qPCR (Fig. 3a). We found that knockdown of FEACR decreased the cell survival rate (Fig. 3b) and induced ferroptosis, as indicated by decreased SLC7A11 and GPX4 protein expression (Fig. 3c and Additional file 1: Fig. S6a, b). In addition, inhibition of FEACR increased MDA levels in cardiomyocytes (Fig. 3d). In isolated neonatal mouse cardiomyocytes, we overexpressed FEACR by infection with an adenovirus expressing FEACR (Additional file 1: Fig. S6c). We found that FEACR attenuated the H/R-induced decline in cell survival (Fig. 3e). In addition, FEACR inhibited H/R-induced ferroptosis, as indicated by the increase in MDA (Fig. 3f) and *Ptgs2* expression (Fig. 3g). We further detected cellular Fe^2+^ and iron deposition by Prussian blue staining and found that FEACR inhibited cellular Fe^2+^ elevation (Fig. 3h) and iron deposition (Additional file 1: Fig. S6d) after H/R. In addition, lipid ROS production was also suppressed by FEACR overexpression in cardiomyocytes (Fig. 3i). These results indicate that FEACR is involved in the regulation of ferroptosis and protects cardiomyocytes from H/R-induced ferroptosis.

### FEACR interacts with and modulates NAMPT protein stability

**Fig. 4:**
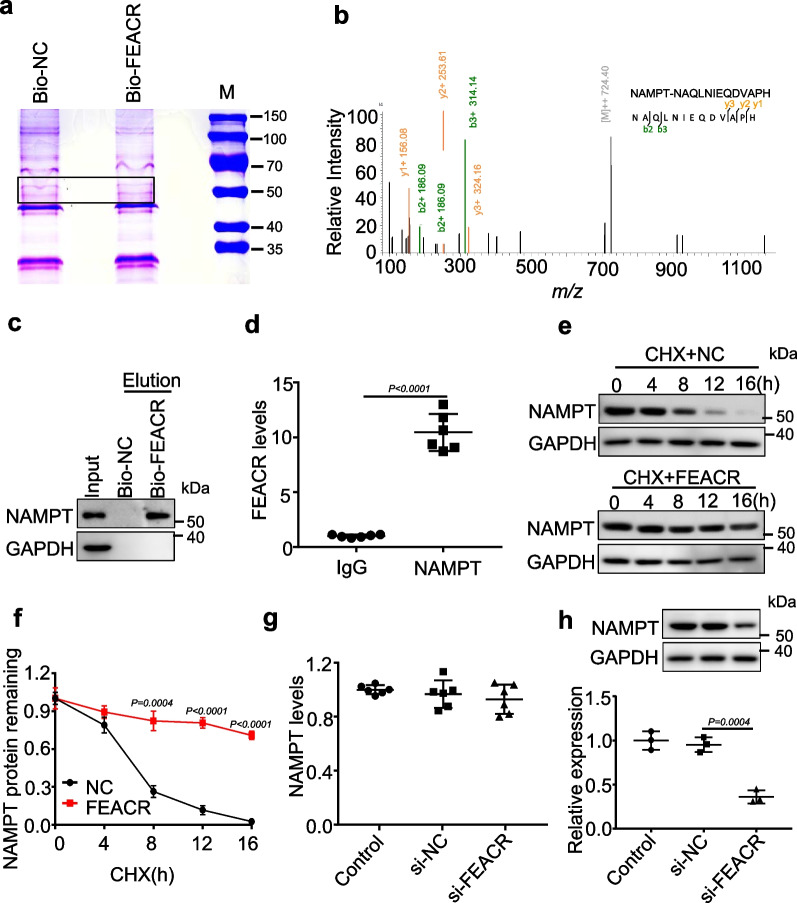
FEACR interacts with NAMPT and maintains its stability. **a** Pull-down assay was performed by using biotinylated FEACR (Bio-FEACR) or negative control RNA (Bio-NC) in cardiomyocytes, and then separated protein molecules by electrophoresis and developed by Coomassie blue staining, proteins from the specific band was used for protein mass spectrometry (MS) identification. **b** NAMPT was identified by LC–MS/MS in the group of biotinylated FEACR. **c** RNA pull-down assay was performed to confirm the NAMPT binding to FEACR by using bio-FEACR or bio-NC. **d** RIP assay was performed by using antibodies against NAMPT to confirm the binding of FEACR to NAMPT by qPCR. **e** 100 μg/mL CHX was used to treat FEACR overexpressed cardiomyocytes at the indicated time. The expression of NAMPT was measured by Western blot. **f** Relative NAMPT protein levels were quantified with Image J. **g** Expression levels of NAMPT mRNA in cardiomyocytes transfected with si-NC or si-FEACR. **h** The expression of NAMPT protein in cardiomyocytes transfected with si-NC or si-FEACR. **a**, **c** n = 3 biological replicates; **d** n = 6 biological replicates; **e**, **f** n = 3 biological replicates; **g** n = 6 biological replicates; **h** n = 3 biological replicates. Data were mean ± SD, and the P*-*value was calculated by One-way ANOVA (**g**, **h**) or student’s t-test (**d**, **f**). The experiment technically repeats three times

We further investigated the molecular mechanisms by which FEACR regulates cardiomyocyte ferroptosis. We performed biotin–streptavidin pull-down assays using biotinylated FEACR in cardiomyocytes and separated the binding protein by electrophoresis (Fig. 4a). We then conducted mass spectrometry (MS) to detect the proteins that specifically bind to FEACR (Additional file 3: Table S2). Among the proteins that were highly enriched in the FEACR pull-down product, we focused on NAMPT for further study because its function in the regulation of ferroptosis was unknown (Fig. 4b). RNA pull-down assays using biotinylated FEACR followed by Western blotting revealed that FEACR bound with NAMPT (Fig. 4c). RNA immunoprecipitation followed by RT‒qPCR verified the interaction between FEACR and NAMPT in cardiomyocytes (Fig. 4d). We then explored the effect of FEACR on NAMPT using cycloheximide (CHX) and found that FEACR overexpression lengthened the half-life of NAMPT after CHX treatment, which indicates that FEACR can maintain the stability of the NAMPT protein (Fig. 4e, f). Furthermore, overexpression of FEACR elevated NAMPT protein levels but did not affect the mRNA levels (Additional file 1: Fig. S7a, b). In contrast, knockdown of FEACR reduced the protein level of NAMPT but did not influence the mRNA levels of NAMPT (Fig. 4g, h). These results indicate that FEACR interacts with NAMPT and preserves the stability of the NAMPT protein.

### NAMPT protects cardiomyocytes from ferroptosis damage

**Fig. 5:**
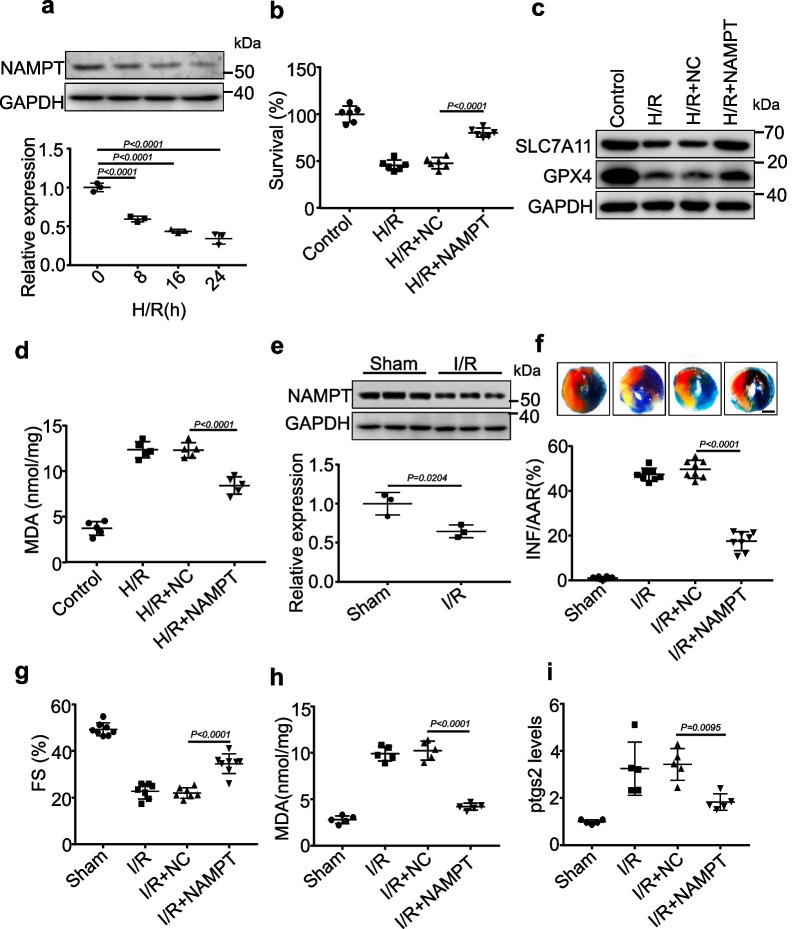
NAMPT inhibits ferroptosis in vitro and in vivo. **a** The level of NAMPT was determined by Western blot in cardiomyocytes treated with H/R at indicated time points. **b**–**d** Cardiomyocytes were infected with adenovirus harboring NAMPT or NC and then were treated with H/R. **b** The cell survival rate was assayed. **c** The ferroptosis-related proteins were detected by immunoblot. **d** The level of MDA was tested. **e** The expression of NAMPT in cardiac tissue from myocardial I/R of mice. **f**–**i** The adenovirus harboring mouse NAMPT or NC was injected into a mouse and subjected to I/R. **f** Evans blue-TTC staining was applied to determine the infarcted zone of the midventricular area, scale bar, 2 mm. **g** FS was determined by transthoracic echocardiographic analysis. **h**, **i** The level of lipid peroxidation MDA and ptgs2 mRNA was measured in the NAMPT overexpressed myocardium upon I/R injury. **a** n = 3 biological replicates; **b** n = 6 biological replicates; **c** n = 3 biological replicates; **d** n = 5 biological replicates; **e** n = 3 mice; **f**, **g** n = 8 mice; **h**, **i** n = 5 mice. Data were mean ± SD, and the P-value was calculated by One-way ANOVA (**a**–**d**, **f**–**i**) or student’s t-test (**e**). The experiment technically repeats three times

To investigate whether NAMPT is involved in H/R- or I/R-mediated ferroptosis, we first detected the expression of NAMPT and found that the expression levels of NAMPT were decreased by H/R (Fig. 5a). The induced expression of NAMPT improved the cell survival rate after H/R exposure (Fig. 5b) and attenuated the downregulation of SLC7A11 and GPX4 in response to H/R (Fig. 5c and Additional file 1: Fig. S7c, d). Moreover, NAMPT inhibited the increase in MDA concentrations after H/R treatment (Fig. 5d). In vivo, the expression of NAMPT was downregulated by I/R surgery (Fig. 5e). We then examined the function of NAMPT in an I/R animal model. We detected the delivery efficiency of adenovirus harboring NAMPT in the myocardium (Additional file 1: Fig. S7e) and found that NAMPT reduced I/R-induced myocardial infarction, as revealed by Evans blue-TTC staining (Fig. 5f, Additional file 1: Fig. S7f). In addition, NAMPT ameliorated cardiac function, as indicated by increased fractional shortening (Fig. 5g). Furthermore, reduced MDA production (Fig. 5h) and decreased mRNA and fluorescence levels of the ferroptosis marker PTGS2 were also observed in I/R mouse hearts overexpressing NAMPT (Fig. 5i, Additional file 1: Fig. S7g). To further investigate whether FEACR regulates ferroptosis via NAMPT, we performed functional gain and loss of FEACR and NAMPT after I/R surgery and H/R treatment in vivo and in vitro. The results showed that NAMPT knockdown inhibited the protective effect of FEACR after I/R (Additional file 1: Fig. S8a), and NAMPT overexpression attenuated the FEACR knockdown-induced increase in *Ptgs2* (Additional file 1: Fig. S8b). Similarly, NAMPT knockdown inhibited the protective effect of FEACR after H/R in cardiomyocytes (Additional file 1: Fig. S8c), and NAMPT overexpression attenuated FEACR knockdown-induced *Ptgs2* increases (Additional file 1: Fig. S8d) in vitro. These data indicate that NAMPT acts as a downstream target of FEACR to participate in the regulation of cardiomyocyte ferroptosis in vitro and in vivo.

### FEACR promotes NAMPT-dependent Sirt1 expression and deacetylation of FOXO1

**Fig. 6:**
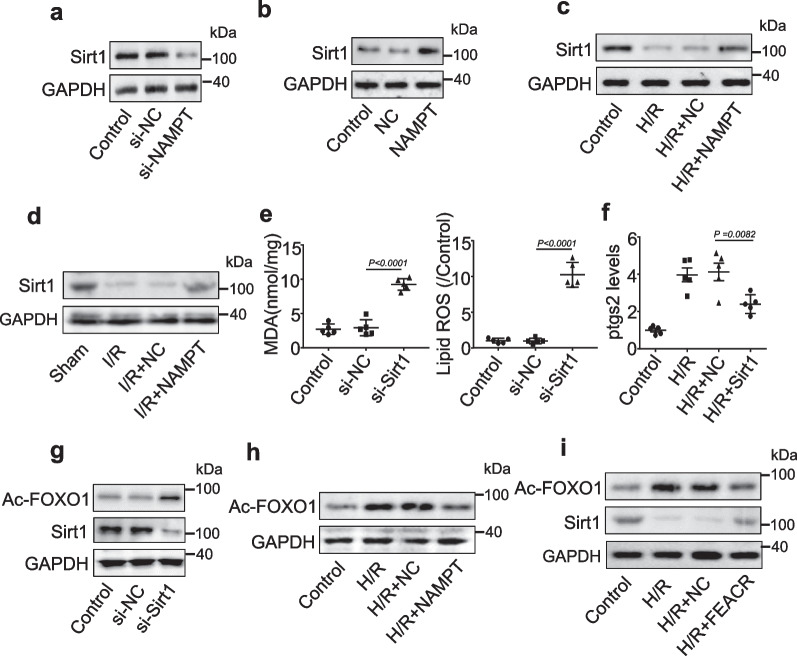
FEACR regulates ferroptosis by the NAMPT-Sirt1-FOXO1 signaling pathway. **a** The protein level of Sirt1 after knockdown of NMAPT evaluated by Western blot. **b** Western blot was performed to test the protein level of Sirt1 upon overexpression of NAMPT. **c** Cardiomyocytes were infected with adenovirus harboring NAMPT or NC and then were treated with H/R. The expression levels of Sirt1 protein were determined by immunoblot. **d** The adenovirus harboring mouse NAMPT or NC was injected into the mouse and subjected to I/R. The expression levels of Sirt1 protein were determined by immunoblot. **e** Cardiomyocytes were transfected with si-Sirt or si-NC, and the levels of lipid ROS and MDA were measured. **f** The expression of ptgs2 mRNA was determined in Sirt1 overexpressed cardiomyocytes followed by H/R treatment. **g** The protein levels of Sirt1 and Ac-FOXO1 were determined by immunoblot in Sirt1-silenced cardiomyocytes. **h** The analysis of protein levels of Ac-FOXO1 was determined by immunoblot in NAMPT-overexpressing cardiomyocytes with H/R treatment. **i** Cardiomyocytes were infected with the adenovirus harboring FEACR or NC and then treated with H/R, the level of Sirt1 and Ac-FOXO1 was determined by immunoblot. **a**–**d**, **g**–**i** n = 3 biological repeats; **e**, **f** n = 5 biological repeats. Data were mean ± SD, and the P-value was calculated by One-way ANOVA. The experiment repeats three times

Next, we explored the downstream signaling of NAMPT. NAMPT plays an important role in the activation of Sirt1, which relies on NAD+ as a substrate [18, 19]. We observed that Sirt1 expression was reduced by NAMPT silencing in cardiomyocytes (Fig. 6a). Consistent with this view, Sirt1 levels were increased in NAMPT-overexpressing cells (Fig. 6b). In addition, we examined the effect of NAMPT on Sirt1 expression in H/R-treated cardiomyocytes or I/R-treated mouse hearts. H/R-induced Sirt1 downregulation was inhibited by NAMPT overexpression (Fig. 6c). The expression of Sirt1 was decreased in the I/R disease model, and the decrease in Sirt1 was suppressed by NAMPT overexpression (Fig. 6d). We then explored the effect of Sirt1 knockdown on ferroptosis. Silencing Sirt1 resulted in increased levels of MDA and lipid ROS in cardiomyocytes (Fig. 6e), while the H/R-induced increase in *Ptgs2* expression was reduced in Sirt1-overexpressing cardiomyocytes (Fig. 6f), indicating that Sirt1 is involved in the regulation of ferroptosis in cardiomyocytes.

FOXO1, a member of the FOXO family, plays critical roles in cell proliferation and differentiation. Sirt1 and FOXO1 interact with each other in response to oxidative stress, and Sirt1 can deacetylate FOXO1 and regulate its transcriptional activity [20]. As expected, silencing Sirt1 induced an increase in the acetylation level of FOXO1 (Fig. 6g). Furthermore, the H/R-induced increase in the acetylation level of FOXO1 was reduced after NAMPT overexpression in cardiomyocytes (Fig. 6h). Overexpression of FEACR in H/R-treated cardiomyocytes inhibited the downregulation of Sirt1 expression and suppressed the elevated acetylation level of FOXO1 (Fig. 6i). In addition, we detected the localization of Sirt1 and FOXO1 and found that FEACR suppressed the H/R-induced decrease in Sirt1 expression in the cytoplasm, which resulted in decreased acetylation of FOXO1 in the cytoplasm (Additional file 1: Fig. S9a, b). Similar results were observed in the nucleus (Additional file 1: Fig. S9c, d). Taken together, these results suggest that Sirt1 and FOXO1 are downstream targets of FEACR and NAMPT in the regulation of cardiomyocyte ferroptosis.

### FEACR-FOXO1 targets FTH1 to inhibit cardiomyocyte ferroptosis

**Fig. 7:**
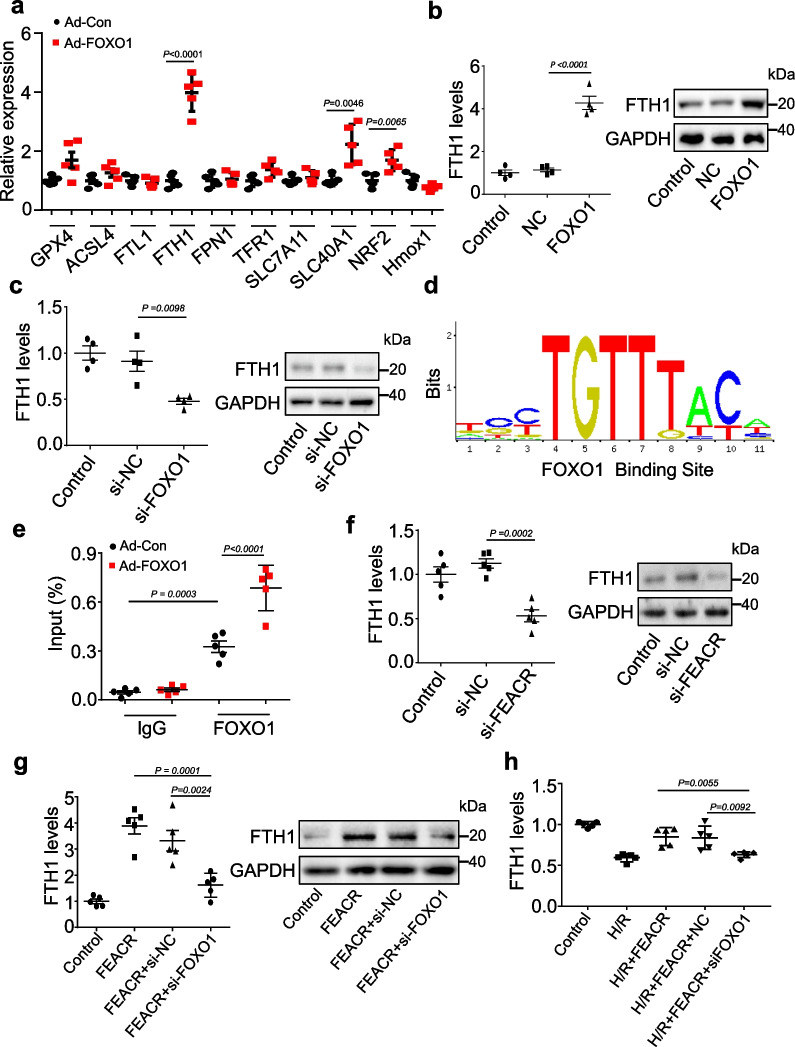
FOXO1 regulates FTH1 expression in cardiomyocytes. **a** Cardiomyocytes were infected with adenovirus harboring FOXO1 or NC, and then the relative expression of genes involved in ferroptosis was determined by qPCR. **b** The expression levels of FTH1 mRNA and FTH1 protein were analyzed by qPCR and immunoblot in cardiomyocytes infected with adenovirus harboring FOXO1 or NC. **c** The expression of FTH1 mRNA and FTH1 protein was analyzed by qPCR and immunoblot in cardiomyocytes transfected with si-FOXO1 or si-NC. **d** Sequence motifs of FOXO1 binding sites within the promoter region of FTH1. **e** ChIP-qPCR assay was performed using antibodies against FOXO1 or IgG in cardiomyocytes infected with adenovirus harboring FOXO1 (Ad-FOXO1) or its negative control (Ad-Con). **f** The mRNA and protein levels of FHT1 were determined by qPCR and immunoblot in cardiomyocytes transfected with si-FEACR or si-NC. **g** Cardiomyocytes were infected with the adenovirus expressing FEACR and NC, co-transfected simultaneously with si-FOXO1 or si-NC. The expression levels of FTH1 mRNA and FTH1 protein were determined. **h** Cardiomyocytes were infected with adenovirus carrying FEACR along with si-FOXO1 or si-NC and then exposed to H/R. The FTH1 mRNA levels were detected by qPCR. **a** n = 5 biological replicates; **b**, **c** n = 4 biological replicates; **e**–**h** n = 5 biological replicates. Data were mean ± SD, and the P-value was calculated by One-way ANOVA. The experiment technically repeats three times

We further identified the downstream targets of FOXO1-dependent regulation of ferroptosis in cardiomyocytes. We tested the expression levels of key regulators associated with ferroptosis. Among them, the mRNA level of *Fth1*, the regulator of iron storage, was increased significantly in FOXO1-overexpressing cardiomyocytes (Fig. 7a). We further overexpressed FOXO1 in cardiomyocytes and found that the mRNA and protein levels of FTH1 were elevated after FOXO1 overexpression (Fig. 7b). Conversely, silencing FOXO1 inhibited the expression of FTH1 mRNA and protein (Fig. 7c). Considering that FOXO1 regulates gene expression through its DNA binding activity, we then examined whether FOXO1 directly targets the *Fth1* gene. A potential FOXO1 binding site was observed in the *Fth1* promoter region (Fig. 7d). We then validated FOXO1 binding to the identified sequence using ChIP‒qPCR and confirmed marked enrichment of the *Fth1* promoter region following FOXO1 overexpression compared to IgG (Fig. 7e). These data indicate that FOXO1 promotes the expression of FTH1 by directly binding to the *Fth1* promoter region through its DNA binding activity.

We next examined the effect of FEACR on the expression of FTH1 and observed that knockdown of FEACR suppressed the expression of FTH1 at the mRNA and protein levels (Fig. 7f), and FEACR overexpression increased FTH1 expression in cardiomyocytes (Additional file 1: Fig. S10a, b). In addition, we explored whether NAMPT participated in the regulation of FTH1 expression. The results showed that induced expression of NAMPT elevated FTH1 expression at both the protein and mRNA levels (Additional file 1: Fig. S10c, d), and knockdown of NAMPT inhibited the mRNA and protein levels of FTH1 (Additional file 1: Fig. S10e, f). Furthermore, silencing FOXO1 attenuated the FEACR-induced increase in FTH1 expression (Fig. 7g), indicating that FEACR regulates FTH1 expression by targeting FOXO1. Overexpression of FEACR inhibited the H/R-induced decrease in FTH1 expression in cardiomyocytes, while knockdown of FOXO1 reversed the effect of FEACR on FTH1 expression (Fig. 7h, Additional file 1: Fig. S10g). It has been reported that FTH1 is a key regulator of ferroptosis, and specific deficiency of FTH1 in cardiomyocytes decreases the level of iron and results in cardiac injury upon aging [21]. We therefore tested whether FTH1 inhibits ferroptosis and cardiac damage after I/R injury. Our results showed that the expression of FTH1 was decreased upon I/R injury (Additional file 1: Fig. S11a). FTH1 overexpression inhibited the I/R-induced SLC7A11 and GPX4 downregulation (Additional file 1: Fig. S11b) and the increase in *Ptgs2* mRNA (Additional file 1: Fig. S11c). We further examined the function of FTH1, and the results showed that overexpression of FTH1 reduced the size of myocardial infarction (Additional file 1: Fig. S11d, e). Next, we explored whether FEACR regulates ferroptosis via FTH1. The results showed that FTH1 knockdown inhibited the protective effect of FEACR after I/R (Additional file 1: Fig. S12a), and FTH1 overexpression attenuated FEACR knockdown-induced *Ptgs2* mRNA upregulation (Additional file 1: Fig. S12b). In addition, FTH1 knockdown inhibited the protective effect of FEACR after H/R treatment in cardiomyocytes (Additional file 1: Fig. S12c), and FTH1 overexpression attenuated FEACR knockdown-induced *Ptgs2* mRNA upregulation (Additional file 1: Fig. S12d, Additional file 4: Table S3) in vitro. These results indicate that FOXO1 and FTH1 are direct downstream molecules of FEACR in the regulation of cardiomyocyte ferroptosis.

## Discussion

Here, we revealed that FEACR, a ferroptosis-associated circRNA, inhibits cardiomyocyte ferroptosis by interacting with NAMPT and targeting the Sirt1-FOXO1-FTH1 pathway. FEACR plays an important role in protecting against I/R-induced dysregulation of the redox state and excessive iron accumulation in a ferroptosis-dependent manner. Mechanistically, FEACR directly interacts with NAMPT and promotes the stability of the NAMPT protein, which results in increased expression of Sirt1, decreased acetylation of FOXO1 and enhanced *Fth1* transcription (Additional file 1: Fig. S13). Therefore, the discovery of the FEACR/NAMPT/Sirt1/FOXO1/FTH1 pathway provides new insight into cardiomyocyte ferroptosis, which is an important contributor to cardiac myocyte loss [22–24]. FEACR can be used as a ferroptosis inhibitor and has the potential for practical applications in severe cardiovascular diseases such as myocardial infarction and cardiac I/R injury.

Emerging studies have implicated the dysregulation of circRNAs in various cardiovascular diseases, and further research on circRNAs that regulate ferroptosis and cardiac I/R is needed [6, 8, 25–28]. Herein, we found that a circRNA named FEACR was downregulated in myocardial tissue after cardiac I/R injury. The rescue of FEACR in cardiac tissues mediated by adenoviral infection inhibits ferroptosis in cardiomyocytes and improves cardiac function. As key regulators of ferroptosis, GPX4 and SLC7A11 are also regulated by FEACR, which indicates that FEACR is involved in ferroptosis after cardiac I/R injury. In addition, FEACR attenuates the main features of ferroptosis, such as *Ptgs2* mRNA upregulation, ROS accumulation, MDA and iron augmentation. It is worth noting that promoting myocardial regeneration is a good way to restore heart function [29]. FEACR might function in different ways under regenerative conditions, and thus, cardiomyocytes are more protected. Perhaps FEACR has no significant effect under regenerative conditions. Although we have confirmed that FEACR protects cardiac functions by inhibiting ferroptosis, whether FEACR also plays an important role in cardiac regeneration under physiological conditions needs to be examined in future studies. Our research provides a new viewpoint for studying the function of circRNAs in cardiomyocyte ferroptosis and a new strategy for treating myocardial infarction and heart failure.

CircRNAs are endogenous covalently closed single-stranded loop RNAs that are generated by back-splicing. Previous studies have revealed that circRNAs regulate gene expression through various molecular mechanisms. For example, circRNA act as miRNA sponges and influence miRNA target genes [30–33]. CircRNA cIARS modulates ferroptosis in hepatic carcinoma by interacting with ALKBH5 [34], and circANRIL modulates ribosomal RNA maturation by circRNA-protein interactions [35]. In the present study, we first proposed that FEACR regulates gene expression by affecting the acetylation of the transcription factor FOXO1 to impact *Fth1* transcription. Our findings provide a novel idea of how the transcriptional activity of *Fth1* is dysregulated in the process of myocardial damage after cardiac I/R. FEACR is mainly located in the cytoplasm where it directly interacts with the NAMPT protein. Induced expression of FEACR maintains the protein level of NAMPT in CHX-treated cardiomyocytes, which indicates that FEACR can maintain protein stability. The expression of FEACR is decreased after I/R injury, which might result in a reduction in NAMPT stability and attenuate the expression of FTH1 to induce ferroptosis in cardiomyocytes. However, NAMPT is an enzyme that catalyzes one step in the biosynthesis of nicotinamide adenine dinucleotide and lacks RNA binding domains. The detailed mechanism by which FEACR binds with NAMPT or whether NAMPT contains RNA binding domains remains to be further explored.

NAD+, a coenzyme of Sirt1 that plays crucial roles in many cellular processes, mediates various redox reactions, including DNA repair, circadian rhythms, cell survival and energy metabolism [36]. The biosynthesis of NAD+ relies on two pathways: the de novo and salvage pathways. Because the adult heart is deficient in rate-limiting enzymes, nearly all cardiac NAD+ is generated from the salvage pathway [37]. NAMPT is a rate-limiting enzyme in the salvage pathway that transfers a phosphoribosyl group to NAM to form NAM mononucleotide (NMN), a precursor of NAD+ [38]. It has also been reported that NAMPT can affect antioxidant protein levels and overall ATP or bioenergetic status [39, 40]. Whether NAMPT plays a protective role by affecting antioxidant proteins or bioenergetics status in I/R injury or ferroptosis remains to be further studied. Sirt1 is an NAD+-dependent histone/protein deacetylase, and we found that the expression level of Sirt1 relied on cellular NAMPT. Knockdown of Sirt1 increases the content of MDA and lipid ROS accumulation, while overexpression of Sirt1 inhibits the increase in *Ptgs2* mRNA, the biomarker of ferroptosis. In addition, the FEACR-dependent increase in NAMPT stability elevates Sirt1 levels, which increases the expression of FTH1 via the deacetylation of FOXO1 and thus promotes the survival of cardiomyocytes.

FOXO1 belongs to the forkhead family of transcription factors, which are characterized by a distinct forkhead domain and a conserved DNA binding domain. FOXO1 contains three positively charged lysine sites: Lys242, Lys245 and Lys262 [41]. The deacetylation of FOXO1 promotes its nuclear localization, and its acetylation means nuclear export [42]. When these lysine sites are acetylated, they no longer have a positive charge and impair the ability of FOXO1 to bind homologous DNA sequences and weaken the transcriptional activity of FOXO1. Ac-FOXO1 can be deacetylated by Sirt1 and Sirt2 and restore its transcriptional activity. The FOXO1 transcription factor has a common DNA binding sequence “TTGTTTAC” for different target genes [43]. However, the function of FOXO1 in cell death, especially its impact on ferroptosis in cardiomyocytes, remains unknown. Therefore, we attempted to screen ferroptosis-related genes regulated by FOXO1 and focused on FTH1 because *Fth1* was the most obviously changed after FOXO1 overexpression. FOXO1 binds with the *Fth1* promoter within the “TGTTTAC” region to enhance *Fth1* transcriptional activity. In this paper, we found that the FEACR-mediated increase in FOXO1 inhibits I/R-induced ferroptosis in cardiomyocytes and myocardial tissue damage by promoting the transcription of *Fth1*, a central molecule involved in iron metabolism in the ferroptosis signaling pathway.

Ferroptosis is a recently recognized iron dependent, nonapoptotic form of cell death that is prevented by iron chelation [22, 44, 45]. Iron overload is an important pathogenic mechanism in several cardiopathic conditions, such as DOX-induced cardiomyopathy or cardiac I/R in animals or patients [46]. Lipid peroxidation produced by the accumulation of Fe^2+^ and ROS leads to ferroptosis. TfR1 couples with Fe^3+^ and controls the uptake of Fe^3+^ outside of the cell and maintains intracellular iron homeostasis [23]. FTH1 is a key regulator of Fe^2+^ storage to control iron metabolism, which can be degraded by NCOA4-mediated ferritinophagy to elevate cellular iron content. FPN is essential for intracellular iron homeostasis in cardiomyocytes to remove excessive iron [47]. FTH1-dependent ferroptosis responds to oxidative stress-mediated myocardial remodeling and heart failure. Therefore, our study suggests the mechanism of the dysregulation of iron homeostasis in cardiomyocyte injury during cardiac I/R. Overall, these results reveal that the FEACR/NAMPT/Sirt1/FOXO1 axis regulates FTH1 and protects cardiomyocytes from ferroptotic cell death.

## Conclusions

Here, we uncovered a ferroptosis-associated circRNA (FEACR), which inhibits ferroptosis in cardiomyocytes by targeting the NAMPT-mediated Sirt1-FOXO1 pathway. Enforced expression of FEACR attenuates ferroptosis of cardiomyocytes caused by H/R and improves I/R-induced myocardial infarction and ameliorates cardiac function. Mechanistically, FEACR directly binds to NAMPT and enhances the protein stability of NAMPT. This leads to the increase in NAMPT-dependent Sirt1, which promotes the transcriptional activity of FOXO1 by reducing its acetylation levels. FOXO1 further upregulates the transcription of *Fth1*, a ferroptosis suppressor, which results in the inhibition of cardiomyocyte ferroptosis. Our findings reveal that the circRNA FEACR-mediated NAMPT/Sirt1/FOXO1/FTH1 signaling axis participates in the regulation of cardiomyocyte ferroptosis and protects the heart against I/R injury. Hence, FEACR and its downstream factors could act as novel targets for alleviating ferroptosis-induced myocardial injury in ischemic heart diseases.

## Supplementary Information

**Additional file 1:** Supplemental figures and methods.

**Additional file 2: Table S1.** List of differentially circRNA in I/R-operated mice heart compared to sham heart.

**Additional file 3: Table S2.** List of biotinylated FEACR versus NC pull-down proteins identified by MS.

**Additional file 4: Table S3.** The primer pairs used for quantitative real-time PCR analysis of gene expression.

## Data Availability

All data generated in the present study may be requested from the corresponding authors.
