# Supplementary material for: Circular RNA FEACR inhibits ferroptosis and alleviates myocardial ischemia/reperfusion injury by interacting with NAMPT
Source: J Biomed Sci. 2023 Jun 27;30:45. doi: 10.1186/s12929-023-00927-1 (PMC10304620; doi:10.1186/s12929-023-00927-1)

SUPPLEMENTAL FIGURES AND METHODS

Isolation of adult cardiomyocytes and fibroblast. The mice subjected to sham and I/R injury and cardiac tissues were used to isolate adult cardiomyocytes. Briefly, "Tyrode solution" consisted of 10 mM taurine (Macklin, T6017), 113 mM NaCl (Sinopharm, 10019318), 10 mM BDM (Sigma, B0753), 4.7 mM KCl (Macklin, P816348), 5.5 mM glucose (Macklin, D823520), 0.6 mM KH_2PO_4 (Macklin, P815662) 10 mM HEPES (Yeasen, 60110ES60), 1.2 mM $MgSO_4$ (Sinopharm, 20025118), 12 mM $NaHCO_3$ (Sinopharm, 10018960) and 10 mM $KHCO_3$(Sinopharm, 20030218). The flow rate of oxygen-rich Tyrode solution was adjusted to 4 mL/min to infuse mouse hearts for 5 min. The digestion solution was Tyrode solution supplemented with 0.5 mg/mL collagenase II (Worthington Biochemical, 42P13894) and 0.16 mg/mL BSA (Solarbio, A8010) perfused at the same flow rate for about 15 min. Then the digested heart was transferred to the stop buffer (Tyrode solution with 1% BSA) and minced with surgical scissors. The isolated cells were filtered through a 100 μm strainer to form a single cell suspension and subsequently collected by centrifugation at 20 g for 3 min at 4 °C. 50% (v/v) percoll in stop buffer was used to re-suspended the cell pellets and centrifuged at 100 g for 5 min at 4 °C. The supernatant is mainly dead cells and fibroblasts which can be used for further study. The pellet is mainly cardiomyocytes, collect them for further study.

CircRNA sequence. The heart tissues derived from sham or I/R-injured mice were frozen in dry ice and then performed circRNA sequence by novogene Inc. The sample

was conducted RNA purification, quantification, and qualification. Library preparation for circRNA sequencing, Clustering and sequencing, and data analysis were executed by Novogene Gene Regulation Department.

RNA interference. We purchased the following siRNA oligonucleotides from GenePharma and the siRNA sequences used were as follows:
si-FEACR, sense 5'-CCCAUUACUCAGCAUGGCATT-3'; antisense 5'-UGCCAUGCUGAGUAAUGGGTT-3'; scramble si-FEACR, sense 5'-UUCUCCGAACGUGUCACGUTT-3'; anti-sense 5'-ACGUGACACGUUCGGAGAATT-3'; si-NAMPT, sense 5'-GGCCAAAUAUUUGUUAGAATT-3'; antisense 5'-UUCUAACAAAUAUUUGGCCTT-3'; scramble si-NAMPT, sense 5'-UUCUCCGAACGUGUCACGUTT-3'; antisense 5'-ACGUGACACGUUCGGAGAATT-3'. si-Sirt1, sense 5'-GUGGCAGAUUGUUAUUAAUTT-3'; anti-sense AUUAAUAACAAUCUGCCACTT. si-FOXO1, sense 5'-UCUUGCAGAAGCUCAGAGCTT-3'; anti-sense GCUCUGAGCUUCUGCAAGATT. Based on the manufacturer's instructions, Lipo2000 Transfection Reagent (Thermo Fisher) was used to transfect siRNAs.

Cell death assay. In the cell death experiment, cardiomyocytes were seeded with 4,000 cells/well in 96 well plates. Hypoxia and reoxygenation (H/R) were

experimented on in cardiomyocytes as previously describe[1]. A cell counting kit purchased from Meilunbio was used to determine the rate of cell survival. Briefly, 100 μL of DMEM/F12 medium containing 10% CCK-8 was added to each well, and after incubation at 37 °C for 1-4 h, the OD value at 450 nm was detected by a microplate reader.

Ferrous iron measurement. The cellular or serum content of iron was detected by a ferrous iron colorimetric assay kit (Elabscience Biotechnology Co., Ltd) after the I/R injury of H/R treatment. A series of ferrous iron standards were added to the plate to make standard curves. For the detection of serum iron, a 200 μL sample was added into the microlon elisa plates and then 100 μL reagent II was mixed in the well incubated for 10 min at 37 °C. Finally, the optical density (OD) value at 593 nm was detected with a microplate reader. In order to detect cellular iron, ten million cardiomyocytes were disrupted in 1 mL reagent I and dissociate for 10 min on the ice. The lysates were centrifuged at 10,000 g, for 10 min. Collect the supernatant to a new tube and used it to detect iron content. The method of cellular iron detection is the same as serum iron detection.

Prussian blue iron staining. The cardiac tissue was fixed in 10% neutral formalin and then dehydrated and embedded. The embedding block was cut 5 μm thick. Soak the section in perls stain and stain for 30 min and rinse fully in distilled water for 5 min. The nuclear fast red solution was used to lightly stain with nuclei for 5 min and

rinsed with tap water for 10 s. Conventionally dehydrated and transparent, seal with resinene. The tissue sections were photographed under a microscope. For cardiomyocytes Prussian blue iron staining, the methods are the same as above.

Immunofluorescence staining. After embedding with OCT (Solarbio, 4583) embedding agent, the heart tissue was frozen sectioned in a freezing microtome with a thickness of 8 μm. The paraformaldehyde was used to fix frozen sections for 20 min, and then wash with PBS for 3 min each, three times. And then incubated with 0.1% Triton for 10 min, and then wash with PBS for 3 min each, three times. Subsequently, the frozen sections were blocked with 2% BSA for 30 min. The primer antibody ptgs2 (Zenbio, 501253, 1:50) and cardiomyocytes marker cTnT antibody (Abcam, ab8295, 1:800) were diluted in 2% BSA. 100 μL diluted primary antibody was added to the frozen sections and incubated for 2 h at room temperature. After that, the section was washed with PBS for 3 min each, three times. The second antibody was diluted in 2% BSA followed by incubation with 1 h. The nuclei were then counterstained with DAPI. The sections were sealed with an antifade solution and observed with laser confocal microscopy.

TUNEL staining. TUNEL staining was performed according to the instructions of the elabscience TUNEL kit (E-CK-A320). Paraffin sections of heart tissue are routinely dewaxed into water. A drop of 100 μL of 1 × protease K working solution was added to each sample and the reaction was performed at 37 °C for 20 min. 100 μL

equilibration buffer with TdT was equilibrated for 10-30 min at 37 °C. Equilibration Buffer with TdT is removed with blotting paper. 50 μL labeled working solution was added to each sample by drop, and the samples were placed in a wet box and reacted at 37 °C for 60 min in the dark. DAPI counterstain and samples were sealed with the antifade solution.

Malondialdehyde (MDA) measurement. The lipid oxidation test kit (Beyotime) uses a color response based on MDA and thiobarbituric acid (TBA) reactions to produce red products to quantitatively detect MDA levels. The experiment was carried out according to the instructions. Take an appropriate amount of standard substance and dilute it with distilled water to 1, 2, 5, 10, 20, and 50 μM, and then use it to make a standard curve. The cardiomyocytes were digested in lysis buffer and determined the content of total protein. Take a clean 1.5 mL centrifuge tube, mix 100 μL sample and 200 μL test work in it, and boil for 15 min. After the mixture is cooled to room temperature, it was centrifuged at 1000 g for 10 min. 200 μL of the supernatant was transferred to a 96-well plate and the OD value at 562 nm was measured. After the MDA content in the solution was calculated according to the standard curve, the MDA content in the sample was expressed by the amount of MDA per unit weight of protein.

C11-BODIPY staining. C11-BODIPY staining is used to detect cellular ROS. For the staining of cardiomyocytes, a total of 10,000 cardiomyocytes were seeded in 24 well

plates before transfection with siRNA or infection with adenovirus and then treated with H/R. For the staining of cardiac tissues, the adenovirus was transfected by intravenous injection and operated on with cardiac I/R injury. 10 μM C11-BODIPY was dropped in the cell or tissue sections and then stained at 37 ℃ for 1 h. Cardiomyocytes were washed three times to remove extra C11-BODIPY. The confocal fluorescence microscope was used to collect the relevant fluorescence signal images, which were excited by 488 nm and 565 nm lasers respectively. When C11-BODIPY is excited by a blue laser (488 nm), the excitation wavelength of the normal cell is 595 nm. Once the lipid of the cell is oxidized, the fluorescence will migrate from 595 nm to 510 nm, which is proportional to the production of lipid oxidation reactive oxygen species.

FEACR identification and polymerase chain reaction (PCR). The cardiomyocytes were digested in buffer GA containing proteinase K at 56 ℃ overnight. The subsequent experiments were performed as instructed by the TIANamp Genomic DNA Kit (Tiangen). The concentration of gDNA was measured by Nanodrop (Thermo Fisher) and 200 ng gDNA as a template was performed PCR. The total RNA was reverse transcription as directed by protocol to cDNA. The sequences of divergent primers of FEACR were 5′-ACCGAGTCTGGTCCATTGCT-3′ (forward) and 5′-GCAGAATCCGATCTCGGCAG -3′ (reverse). The sequence of convergent primers of FEACR were 5’- TCAGATGCCTTGGATGCAGCTA-3’ (forward) and 5’-CCTCCTGCACTGACGATGTGGG -3’ (reverse). The sequences of divergent

primers of GAPDH were 5'- AAGAAGGTGGTGAAGCAGGCAT-3' (forward) and 5'-GACGAGGAAACACTCTCCTGAG-3' (reverse). The sequence of convergent primers of GAPDH were 5'- GATGGGTGTGAACCACGAGAAA-3' (forward) and 5'- GCCCCACGGCCATCACGCCACA-3' (reverse). The PCR production was electrophoresis in 2% agarose gel. FEACR and FBXW4 derive from the same transcript. We use the splice sites of FEACR to distinguish the PCR products of FEACR and FBXW4 primers. More specifically, the product of FEACR PCR primers contains the splice sites of FEACR, while the product of FBXW4 PCR primers does not.

Quantitative reverse transcription-PCR (qRT-PCR). Transgene's one-step gDNA removal and cDNA synthesis kit was used to synthesize cDNA from total RNA extracted with Trizol reagent (Vazyme). To perform the real-time quantitative PCR, 0.2 μM primers and 1X SYBR green mix were used (Supplementary Table S3), and 50 ng cDNA sample. Mouse GAPDH was used as internal control genes. Amplification and quantitative measurement were performed using the QuanStudio3 PCR system (Thermo Fisher) by the $2^{-\Delta\Delta CT}$ method.

Myocardial injury size measurement. We used Evans blue (Sigma-Aldrich) and 2,3,5-Triphenyl-tetrazolium chloride (TTC, Sigma-Aldrich) staining to determine the size of myocardial injury. After I/R treatment, the mice were injected with 1% Evans blue form ventriculus sinister until the limbs were blued. Take the heart out of the

body and froze at -80 °C for 15 min and then the heart was cut into 0.2 cm thick slices. The slice was stained by 2% TTC at 37 °C for 30 min and avoided light. The photo was captured by a digital camera (Nikon) and the areas of the non-ischemic left ventricle (LV), area of at risk (AAR), and infarction area (INF) were quantitative by Image J.

Lipo2000 transfection. The process of transfection follows instructions. Primary cardiomyocytes were seeded with 100,000 cells/well in a six-well plate. Tube A: 20 pM siRNA was dissolved in 50 μL no-serum opti-MEM. Tube B: 1 μL lipo2000 was dissolved in 50 μL opti-MEM. Tube C: mix tubes AB and stand at room temperature for 20 min. Add tube C to the six-well plate and change the culture medium 6-8 h later.

Echocardiographic assessment. Mice were treated with I/R, and the cardiac function was monitored by echocardiogram using a visual sonics vevo2100 ultrasound imaging system. The system has two models: B-model and M-model, M-model is used to measure left ventricular ejection fraction (EF), fractional shortening (FS), LV posterior wall (LVPW), and LV internal diameter (LVID). The echocardiographic assessments were blinded, and at least three beats were averaged for all measurements.

RNA binding protein immunoprecipitation (RIP) assay. RIP was performed as

previously described[2]. Cardiomyocytes were lysed in lysis buffer (150 mM NaCl, 0.5 mM dithiothreitol, 0.1% SDS, 1% NP-40, 50 mM Tris buffer, pH 7.4, 1 mM EDTA, 1 mM phenylmethyl sulfonyl fluoride, 0.5% sodium deoxycholate) supplemented with 1x proteinase inhibitor cocktail, a 30 min period was spent on the ice. The lysed product was centrifuged at 12,000 rpm, 4 °C for 20 min, and aliquoted 50 μL lysed product as input. The remaining lysates were incubated with rabbit IgG negative control and NAMPT antibody respectively overnight. The next day, the lysates mixture was furtherly incubated with protein A/G agarose beads for 4 h. After washing beads with lysis buffer five times, the beads-RNA-protein was extracted by Trizol reagent and analyzed by qRT-PCR.

Nuclear/cytosol fractionation. A nuclear/cytoplasm fractionation kit has been invented to separate the nuclei and cytoplasm. The cell was collected and lysed by cytosol extraction buffer A (CEB-A) and CEB-B and centrifuged at 16,000 g, 5 min, 4 °C. Transfer the supernatant (cytoplasm) into a pre-chilled EP tube. The pellet was resuspended by nuclear extraction buffer mix and centrifuged at 16,000 g, 10 min, 4 °C. Transfer supernatant (nuclear) to a pre-chilled EP tube. Cytoplasm and nuclear fraction were used to identify the expression of FEACR in different cellular components.

Fluorescence in situ hybridization (FISH). The cellular distribution of FEACR is examined by FISH staining. The process of FISH staining was guided by instructions

of GenePharma Co., Ltd. Paraformaldehyde was used to fix for 15 min, followed by permeation with 0.1% buffer A for 15 min. The blocking solution was incubated for 30 min. Discard the blocking solution and add 2x buffer C, incubate at 37 ℃ for 30 min. 1 μM cy3-FEACR was denaturalized at 75 ℃ for 10 min and mixed with SA-Cy3 and PBS with volume ratio 1:1:8. 10 μL of the prepared probe working solution was mixed with 90 μL of buffer E, added to wells, and hybridized overnight at 37 ℃. The hybridization solution was discarded and washed with buffer F and buffer C, respectively on the next day. cTnT and DAPI were counterstained, and the anti-quench agent was added and observed under confocal microscopy. The sequence of FEACR FISH probe: TGGAATGCCTGCCATGCTGAGTAAT.

Chromatin Immunoprecipitation (ChIP). The ChIP experiment was performed according to the manufacturer's protocol using a ChIP assay kit (Millipore, Billerica, MA). Briefly, 1% formaldehyde was used to crosslink the mouse cardiomyocytes at room temperature for 15 min and terminated the crosslinking reaction with ice-cold 0.125 M glycine for 5 min. Then the cells were centrifuged at 2,500 g for 3 min and resuspended in ice-cold cell lysis buffer. The chromatin fragments with an average length of about 400–800 bp were obtained by sonicating the cell lysate and the samples were precleared using protein A-agarose (Roche) for 2 h at 4 °C followed by overnight incubation with anti-FOXO1 or anti-mouse IgG at 4 °C. The immune complexes were precipitated with protein A-agarose for 6 h. The precipitated DNA fragments were purified using a QIAquick Spin Kit (Qiagen) and the FTH1 promoter

was detected using the following primer pairs: 5′-TACTCACTCGCATTCTTTCCA-3′ (forward) and 5′-CCATGCTTGTTAAATACATCCC-3′ (reverse).

SUPPLEMENTAL FIGURE LEGENDS

Supplementary Figure 1. Identification of circRNAs in cardiomyocytes. (a, b) The protein levels of SLC7A11 and GPX4 after I/R surgery at the indicated time were examined by immunoblot and quantified by image J. **(c)** The expression levels of top 10 upregulated circRNAs selected from RNA-seq data were determined by qPCR in cardiomyocytes with H/R treatment. **(d)** The representative agarose gel image shows that FEACR was amplified by divergent primers from cDNA but not gDNA. **(e)** The expression of FEACR in adult cardiomyocytes or fibroblast after I/R injury or sham. **(f)** Representative images of FISH with junction-specific probes of FEACR indicate its subcellular localization. Red represents FEACR, cTnT is used to label cardiomyocytes, and DAPI labels nuclei.

Supplementary Figure 2. FEACR improves cardiac functions after I/R injury. (a) The ratio of AAR/LV refers to the level of the risk area. **(b-f)** The left ventricular ejection fraction (EF), LV internal diameter at end-diastole (LVIDd), LV internal diameter at end-systole (LVIDs), left ventricular posterior wall at end-diastole (LVPWd), and left ventricular posterior wall at end-systole (LVPWs) was monitored by the transthoracic ultrasonic imaging system.

Supplementary Figure 3. FEACR attenuates ferroptosis *in vivo.* (a) Representative fluorescence images showing the ptgs2 (red) counterstained with

cardiomyocytes marker cTnT (green) and DAPI (blue) in the left panel. Statistical analysis of the proportion of the ptgs2-positive cells in each group in the right panel. **(b, c)** The level of ptgs2 mRNA and MDA after I/R injury for the indicated time in NC and FEACR overexpressed mice.

Supplementary Figure 4. FEACR has no impact on non-ferroptotic cell death. (**a, b)** FEACR is dispensable for the expression of necroptosis proteins RIPK1, RIPK3, and MLKL after cardiac I/R. **(c, d)** FEACR does not affect apoptosis as indicated by the protein levels of BAX. **(e, f)** The representative images and quantitative analysis of TUNEL in the cardiac tissues.

Supplementary Figure 5. H/R induces ferroptosis in cardiomyocytes. (a) Cardiomyocytes pretreated with Ferrostatin-1 (Fer-1) or DMSO for 2h and then treated with H/R at the indicated time points, the cell survival rate was determined by CCK-8. **(b)** Cardiomyocytes pretreated with Fer-1 and then treated with H/R, the level of lipid peroxide MDA was assayed. **(c)** Cardiomyocytes pretreated with Fer-1 and then treated with H/R, the expression levels of SLC7A11 and GPX4 were detected by immunoblot.

Supplementary Figure 6. FEACR is involved in H/R-induced ferroptosis. (a, b) Quantitative statistics analysis of protein expression of SLC7A11 and GPX4 after FERCR inhibition in cardiomyocytes. **(c)** The FEACR expression was detected by

qPCR in cardiomyocytes infected with adenovirus harboring FEACR. **(d)** The accumulation of ferric ions was examined by Prussian blue staining in cardiomyocytes.

Supplementary Figure 7. FEACR elevates the expression of the NAMPT protein. (a) The expression of FEACR was detected by qPCR in cardiomyocytes infected with adenovirus harboring FEACR and NC. **(b)** The expression of NAMPT was detected by Western blot in cardiomyocytes infected with adenovirus harboring FEACR and NC. **(c, d)** Image J quantified the Western blot results of SLC7A11 and GPX4 expression after H/R treatment in NC and NAMPT overexpressed cardiomyocytes. **(e)** The adenovirus vector expressing NAMPT was delivered into the mice and the expression level of NAMPT was measured by qPCR *in vivo*. **(f)** Quantification of the left ventricle risk size measured as the percentage of left ventricle area (LV) and area at risk (AAR). **(g)** The percentage of ptgs2 positive tissue was calculated from the immunofluorescent staining experiment.

Supplementary Figure 8. NAMPT rescues FEACR knockdown-induced ferroptosis. (a) The mice were delivered with adenovirus harboring with FEACR, si-NAMPT, and subjected to I/R injury, the expression of ptgs2 was detected by qPCR. **(b)** The mice were delivered with adenovirus harboring si-FEACR and NAMPT to detect the expression of ptgs2. **(c)** Cardiomyocytes were infected with adenovirus harboring with FEACR or si-NAMPT and then exposed to H/R. The

mRNA levels of ptgs2 were measured by qPCR. **(d)** Cardiomyocytes were co-transfected with adenovirus harboring with siFEACR or NAMPT, and the ptgs2 expression was detected by qPCR.

Supplementary Figure 9. FEACR regulates the cellular distribution of FOXO1 after H/R treatment. (a, b) The expression of FOXO1, Ac-FOXO1, and Sirt1 in the cellular cytoplasm was examined by immunoblot and quantified by image J. **(c, d)** The nuclear FOXO1, Ac-FOXO1, and Sirt1 were examined by immunoblot and quantified by image J.

Supplementary Figure 10. Both FEACR and NAMPT regulate the expression of FTH1. (a, b) Cardiomyocytes were infected with adenovirus harboring FEACR or NC. The mRNA levels of FTH1 were detected by qPCR. The protein levels of FTH1 were detected by immunoblot. **(c, d)** The mRNA and protein levels were determined by qPCR and immunoblot in cardiomyocytes infected with adenovirus harboring NAMPT or NC. **(e, f)** The mRNA and protein levels were detected by qPCR and immunoblot in cardiomyocytes transfected with si-NAMPT or si-NC. **(g)** Cardiomyocytes were co-transfected with FEACR and si-FOXO1 and subjected to H/R treatment, the expression of FTH1 was detected by immunoblot and quantified by image J.

Supplementary Figure 11. FTH1 regulates ferroptosis and attenuates cardiac

damage. (a) The immunoblot was used to examine the expression of FTH1, FOXO1, Ac-FOXO1, and Sirt1 after I/R injury. The expression levels were quantified by image J. **(b)** The SLC7A11 and GPX4 were examined by immunoblot and quantified by image J. **(c)** The expression of ptgs2 was examined by qPCR in FTH1-overexpressed mice subjected with I/R injury. **(d, e)** The representative images exhibit Evans blue-TTC stain of heart sections, Scale bar, 2mm. Representative images and quantitative data for infarct size (INF) and relative area at risk (AAR) in heart sections from mice subjected to I/R injury and treated with adenoviruses containing FTH1 genes.

Supplementary Figure 12. FTH1 restrains FEACR knockdown-induced ferroptosis. (a) The mice were delivered with adenovirus harboring with FEACR, si-FTH1, and subjected to I/R injury, the expression of ptgs2 was detected by qPCR. **(b)** The mice were delivered with adenovirus harboring with si-FEACR and FTH1 to detect the expression of ptgs2. **(c)** Cardiomyocytes were infected with adenovirus harboring FEACR and si-FTH1 and then exposed to H/R. The mRNA level of ptgs2 was measured by qPCR. **(d)** Cardiomyocytes were co-transfected with adenovirus harboring with si-FEACR and FTH1 and the ptgs2 expression was measured by qPCR.

Supplementary Figure 13. Graphic abstract of FEACR function in ferroptosis signaling. FEACR involves in the regulation of cardiac ferroptosis via

NAMPT/Sirt1/FOXO1/FTH1 pathway. FEACR stabilizes NAMPT and subsequently elevates Sirt1 expression, which leads to the deacetylation of Ac-FOXO1. Sirt1-mediated deacetylation results in the elevation of FOXO1 and thereby promotes FTH1 transcription and inhibits ferroptosis. In a disease model, the expression of FEACR is decreased after I/R, which destruct the steady state and leads to initiate ferroptosis.

REFERENCES

[1] GAO XQ, LIU CY, ZHANG YH, et al. The circRNA CNEACR regulates necroptosis of cardiomyocytes through Foxa2 suppression [J]. Cell Death Differ, 2022,29(3): 527-539.

[2] LIU CY, ZHANG YH, LI RB, et al. LncRNA CAIF inhibits autophagy and attenuates myocardial infarction by blocking p53-mediated myocardin transcription [J]. Nat Commun, 2018,9(1): 29.

Supplementary Figure 1

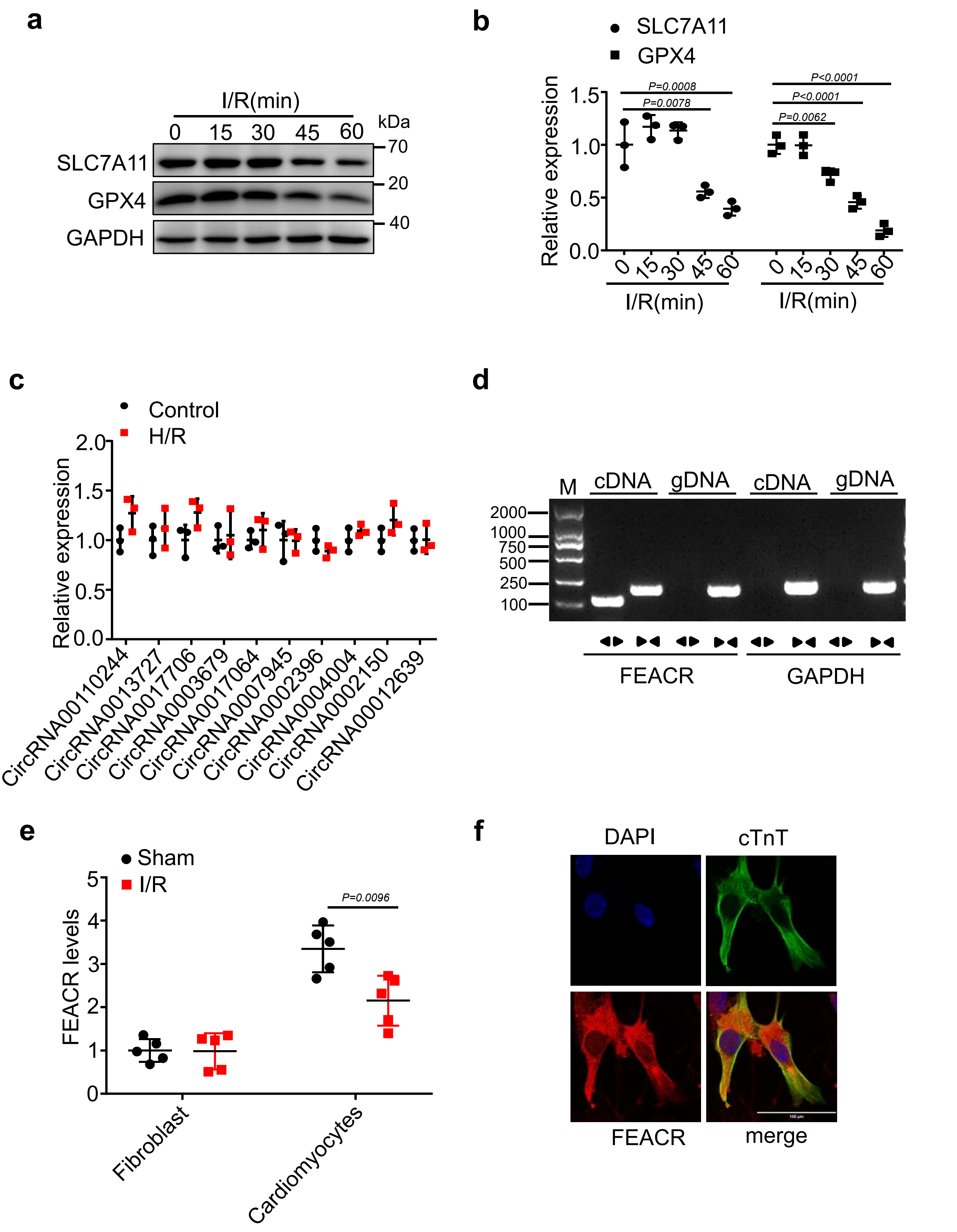

Supplementary Figure 2

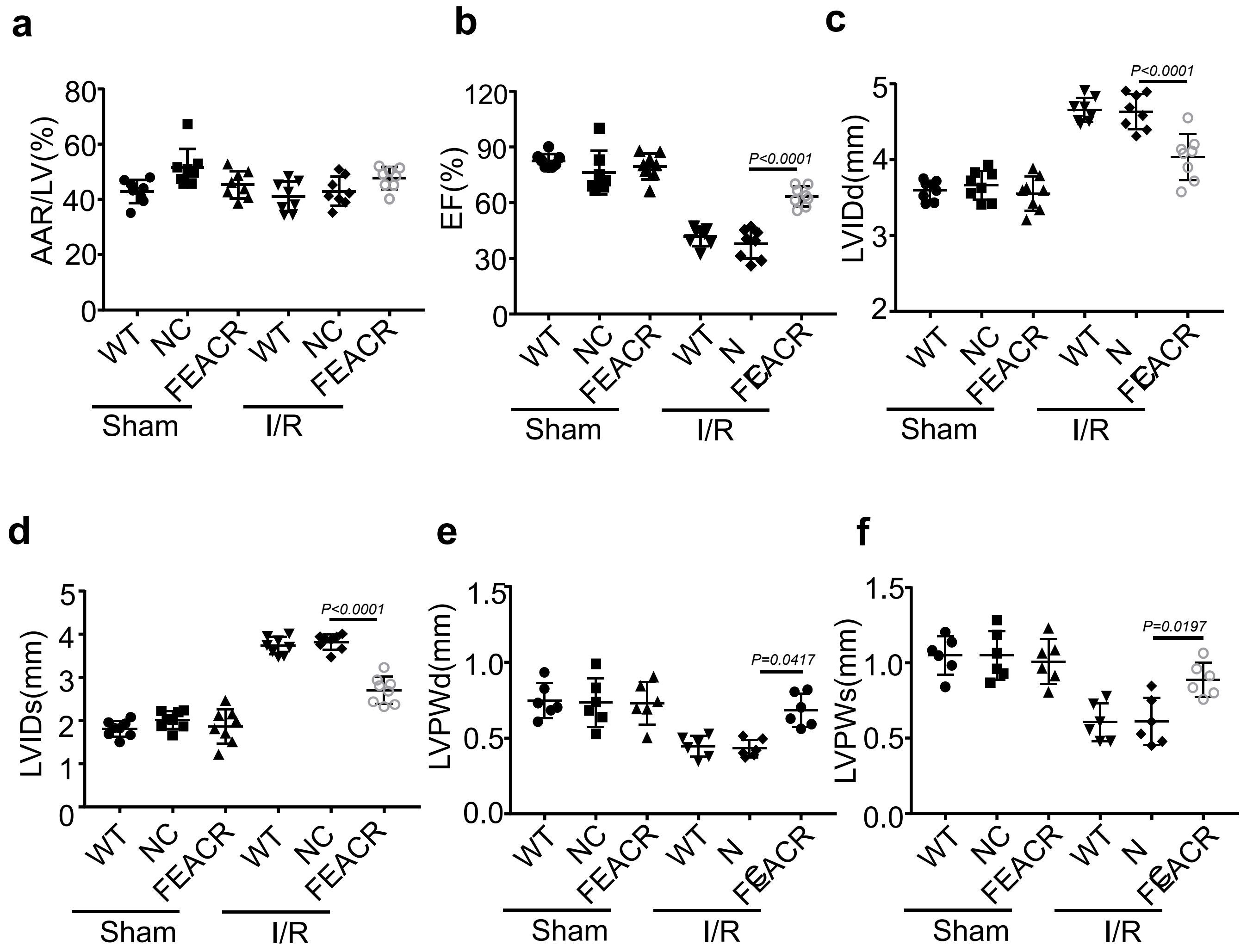

Supplementary Figure 3

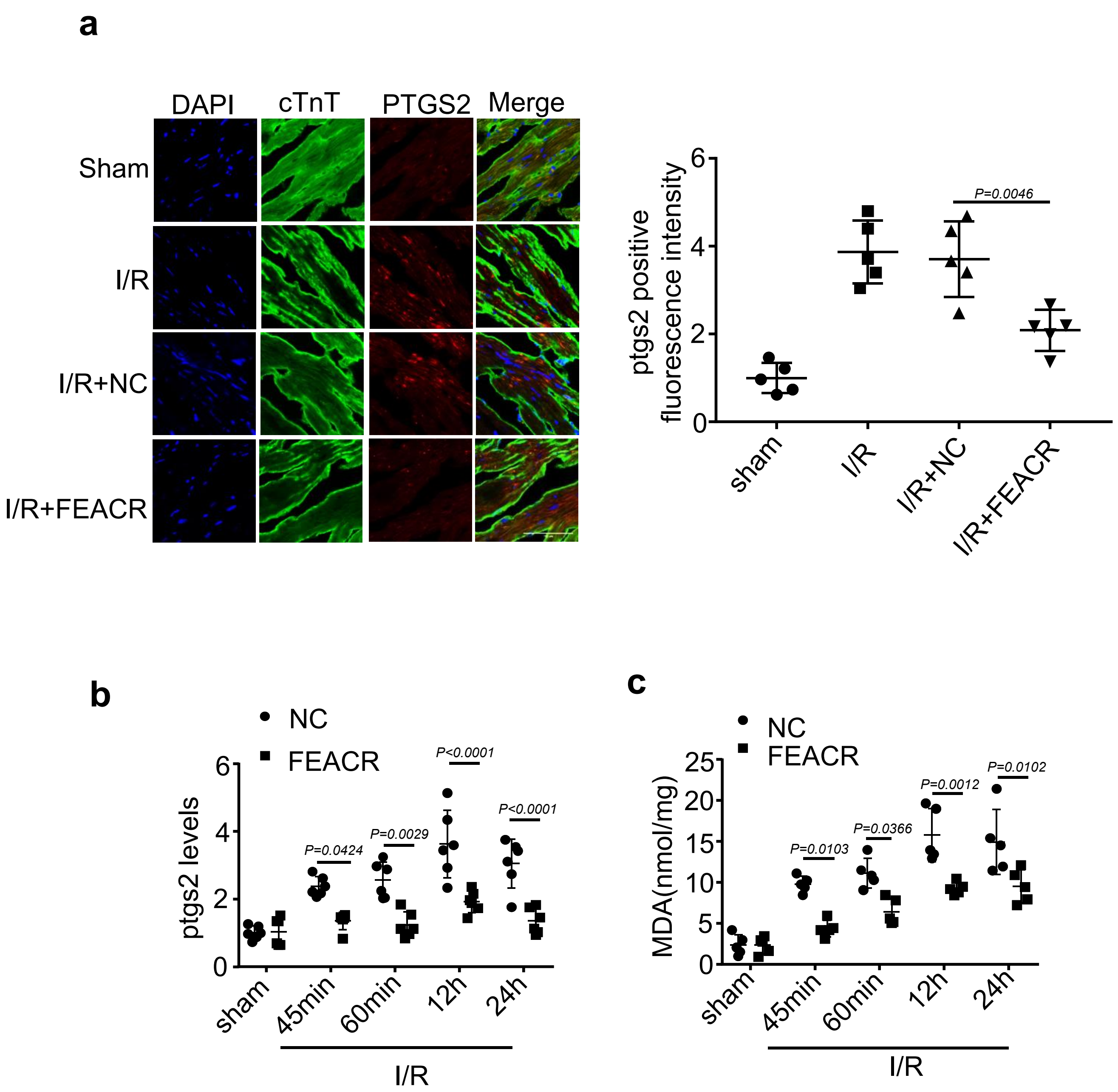

Supplementary Figure 4

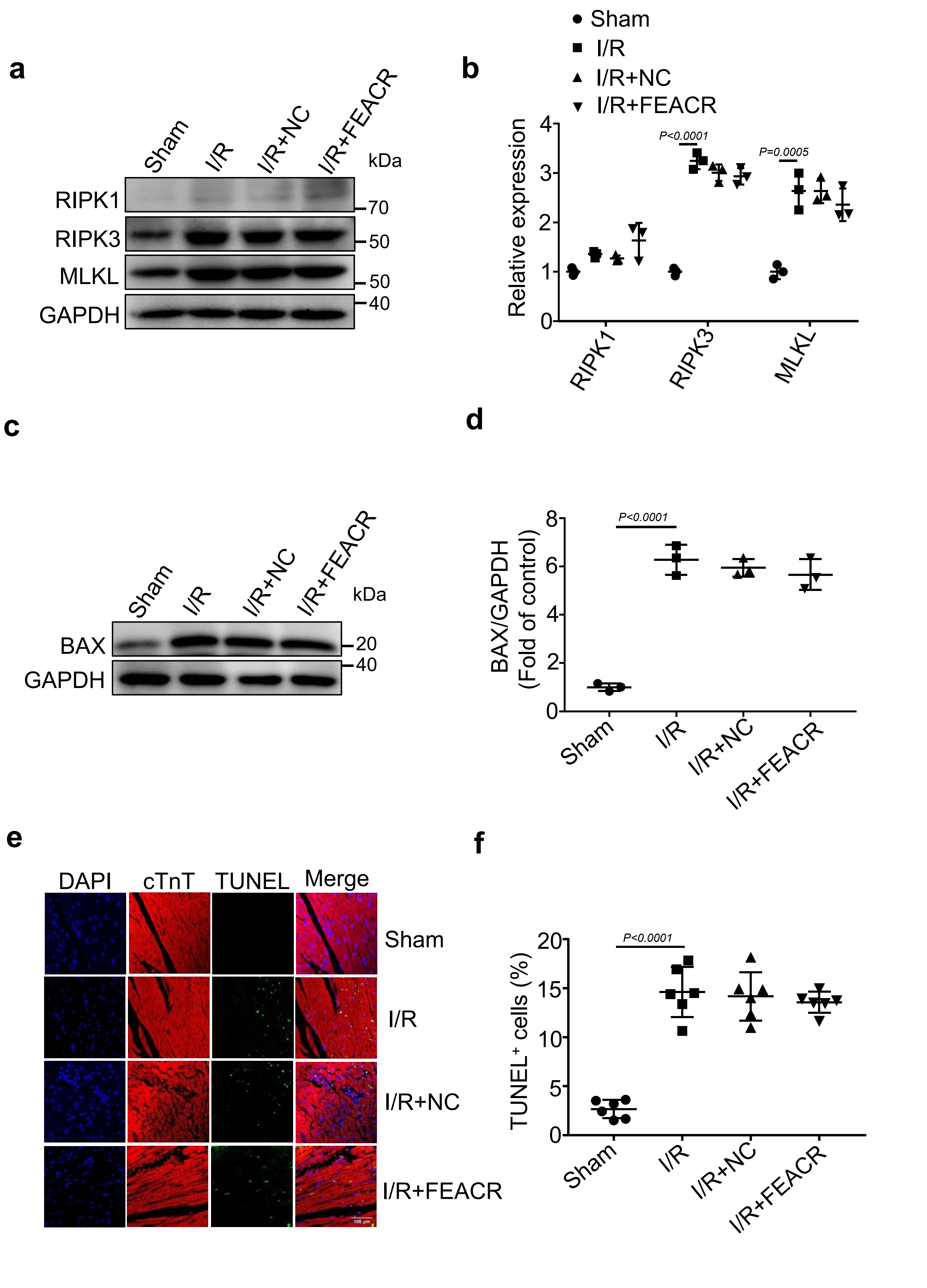

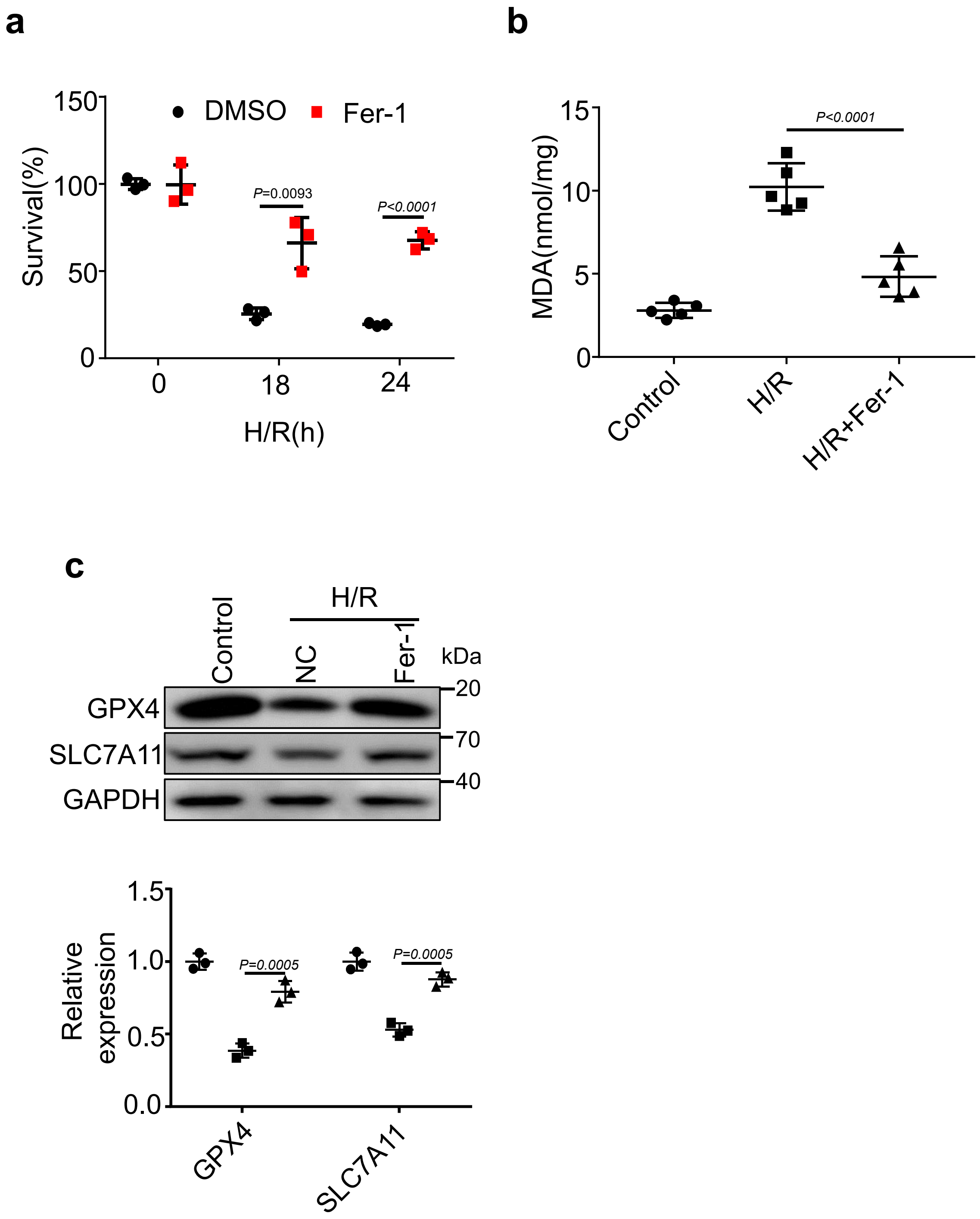
a
150
100
50
0
Survival(%)
DMSO
Fer-1
P=0.0093
P<0.0001
0
18
24
H/R(h)
b
15
10
5
0
MDA(nmol/mg)
P<0.0001
Control
H/R
H/R+Fer-1
c
H/R
Control
NC
Fer-1
kDa
GPX4
SLC7A11
GAPDH
20
70
40
1.5
1.0
0.5
0.0
Relative expression
P=0.0005
P=0.0005
GPX4
SLC7A11

Supplementary Figure 6

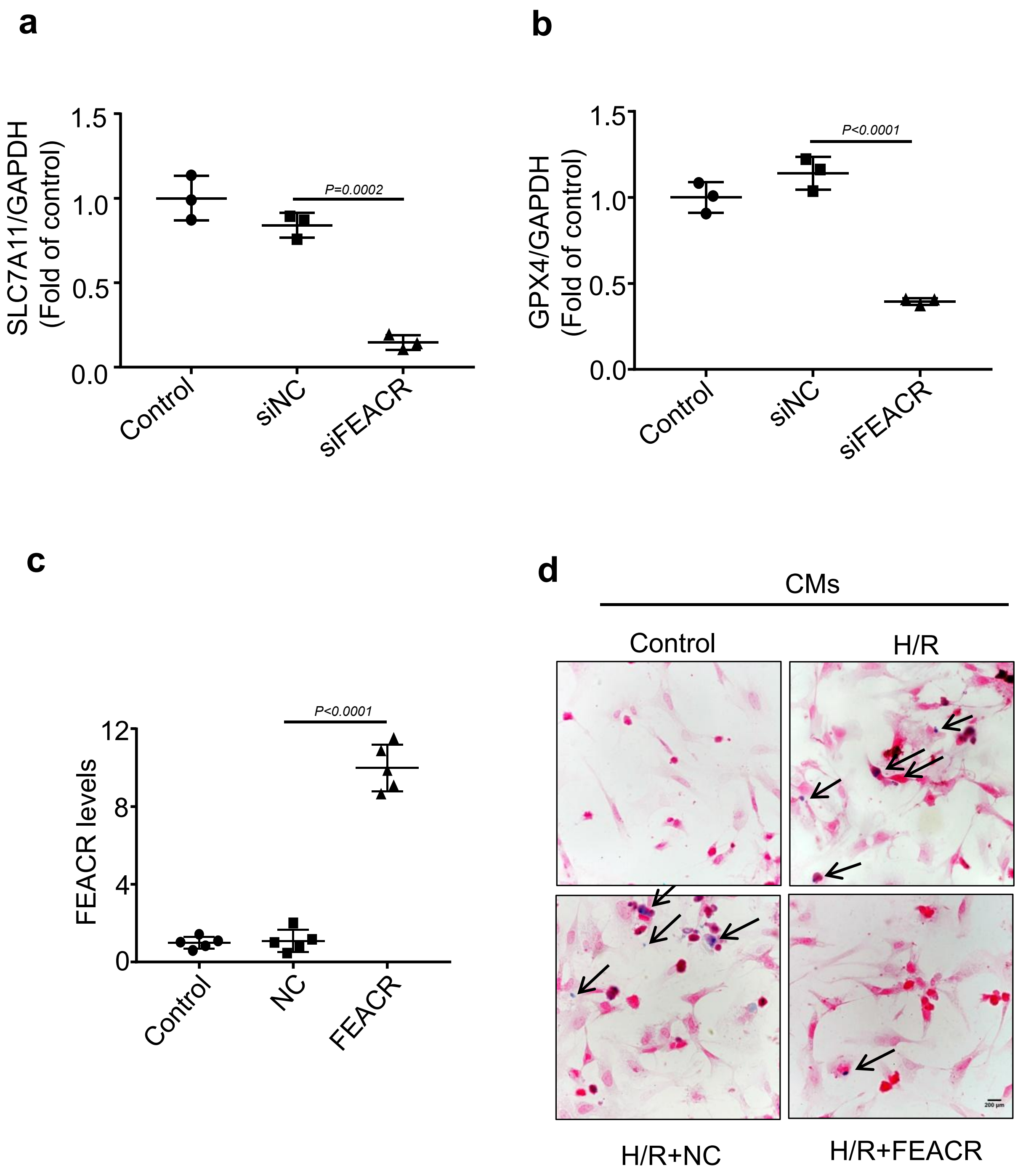

Supplementary Figure 7

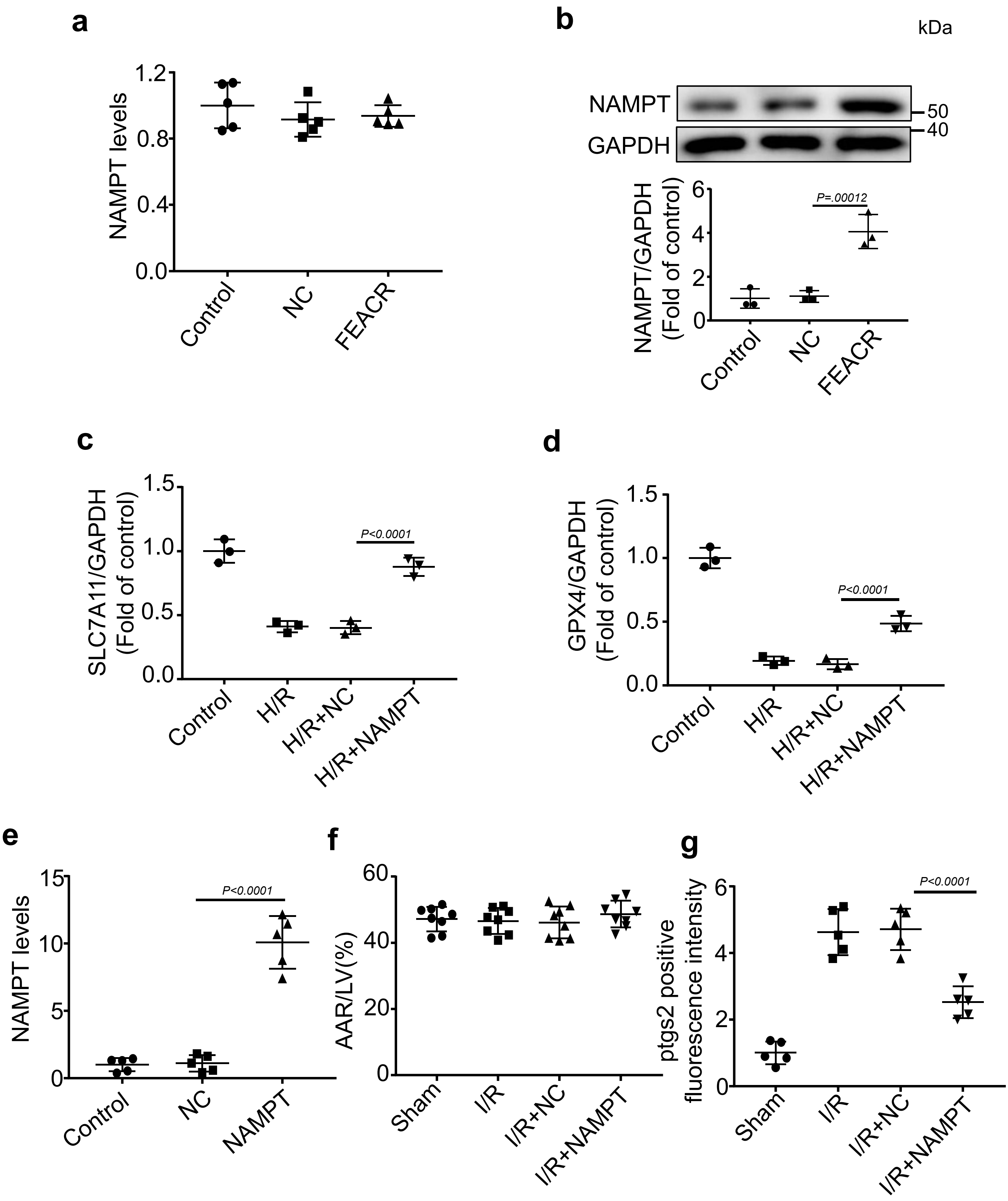

Supplementary Figure 8

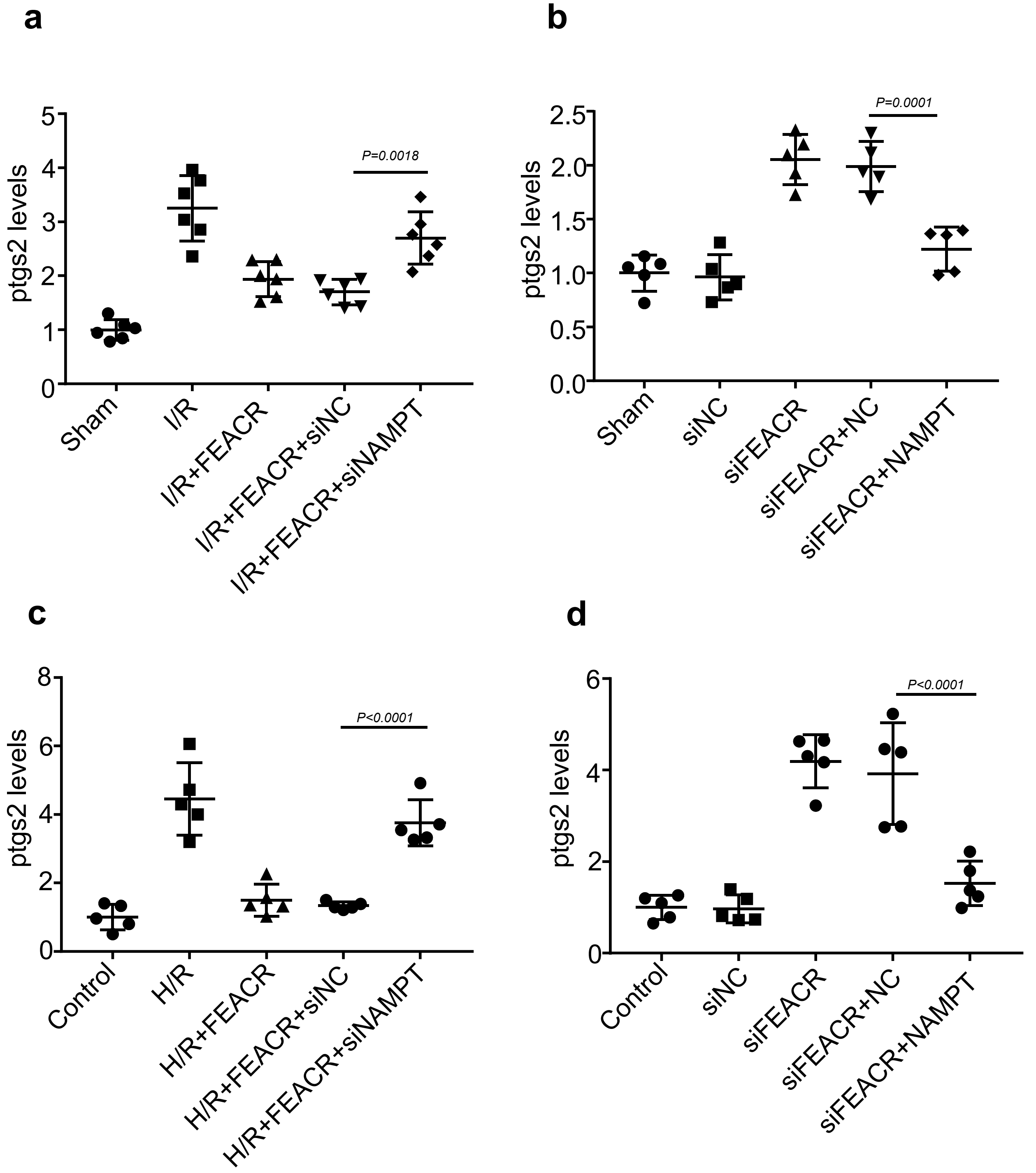

Supplementary Figure 9

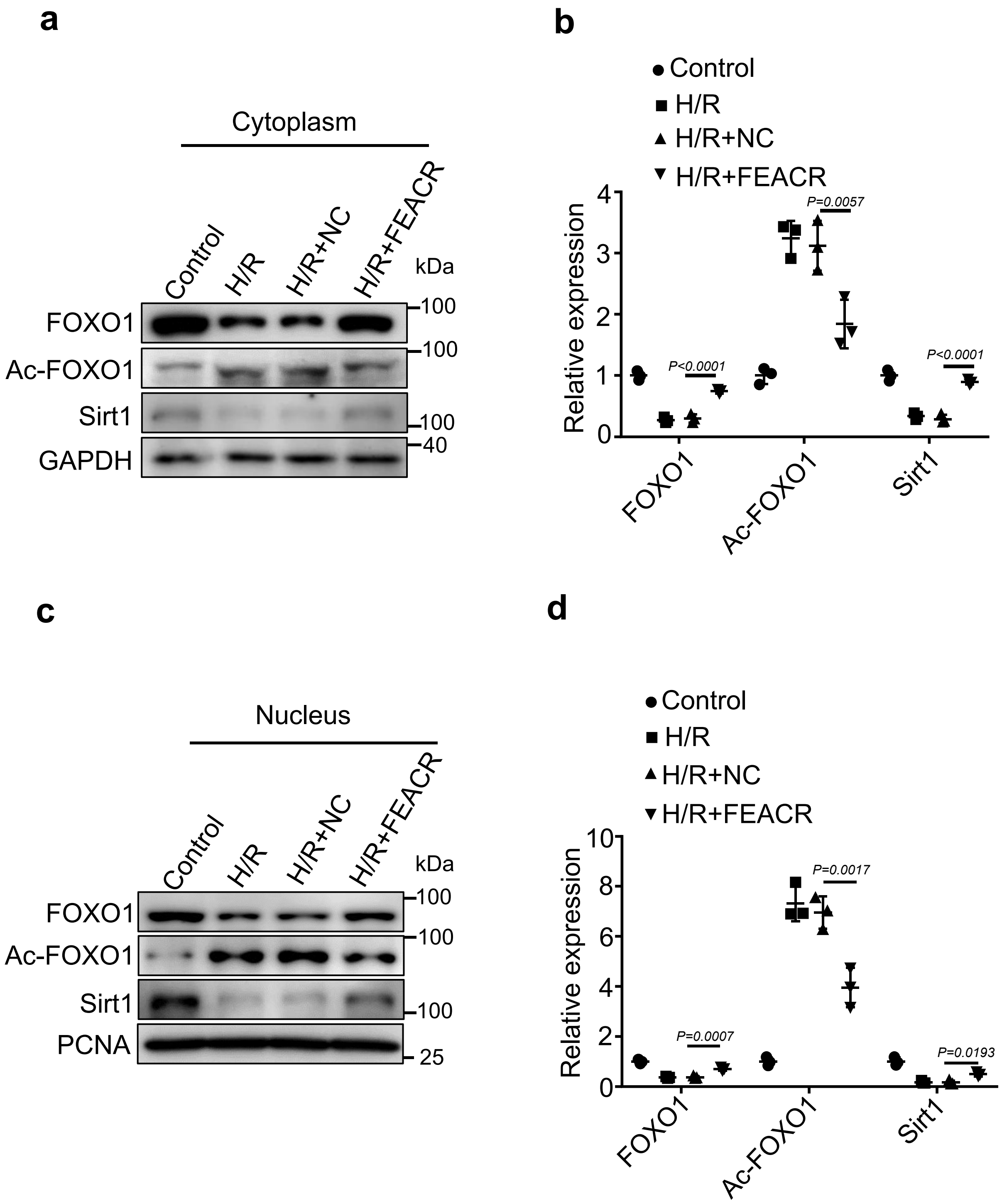

Supplementary Figure 10

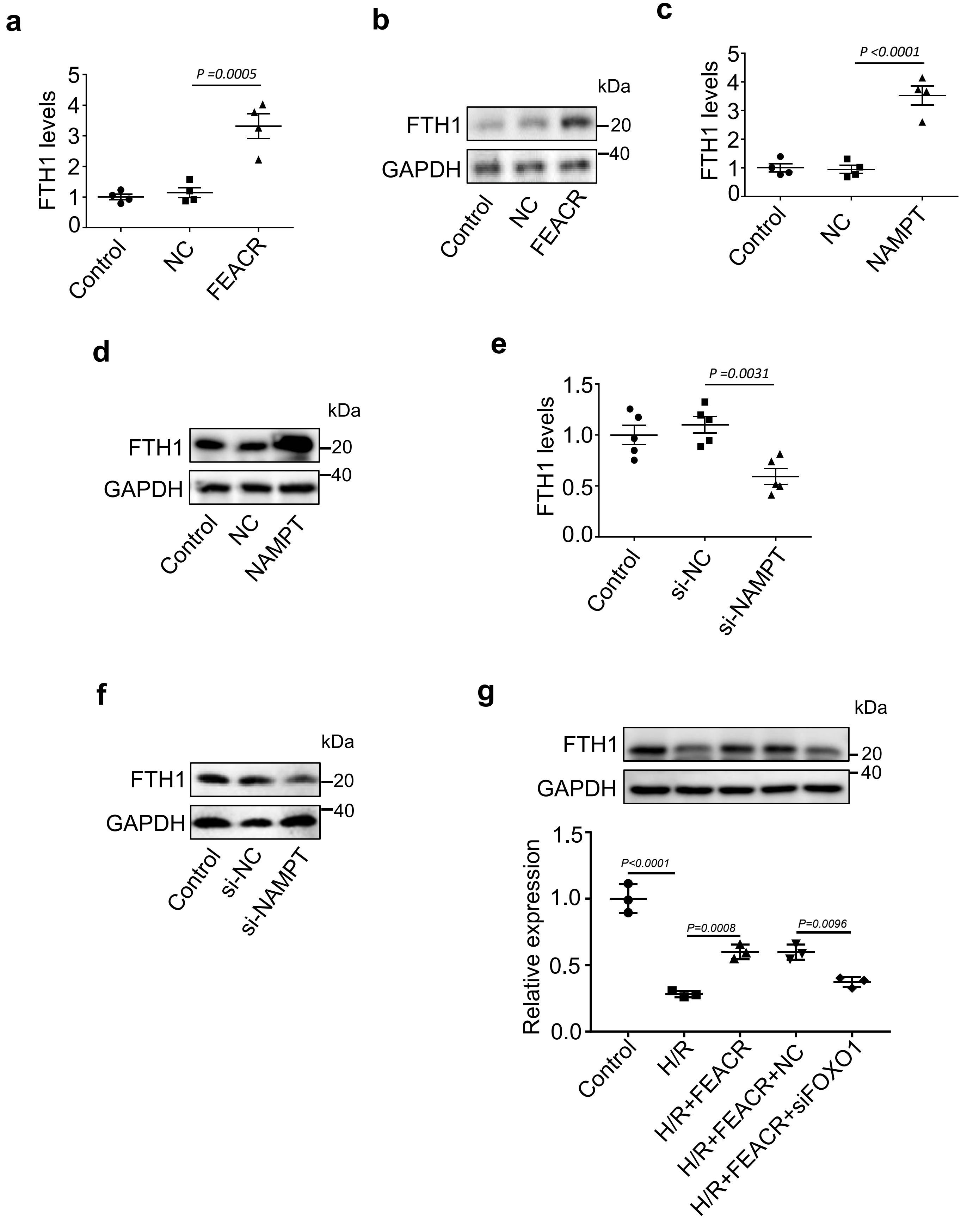

Supplementary Figure 11

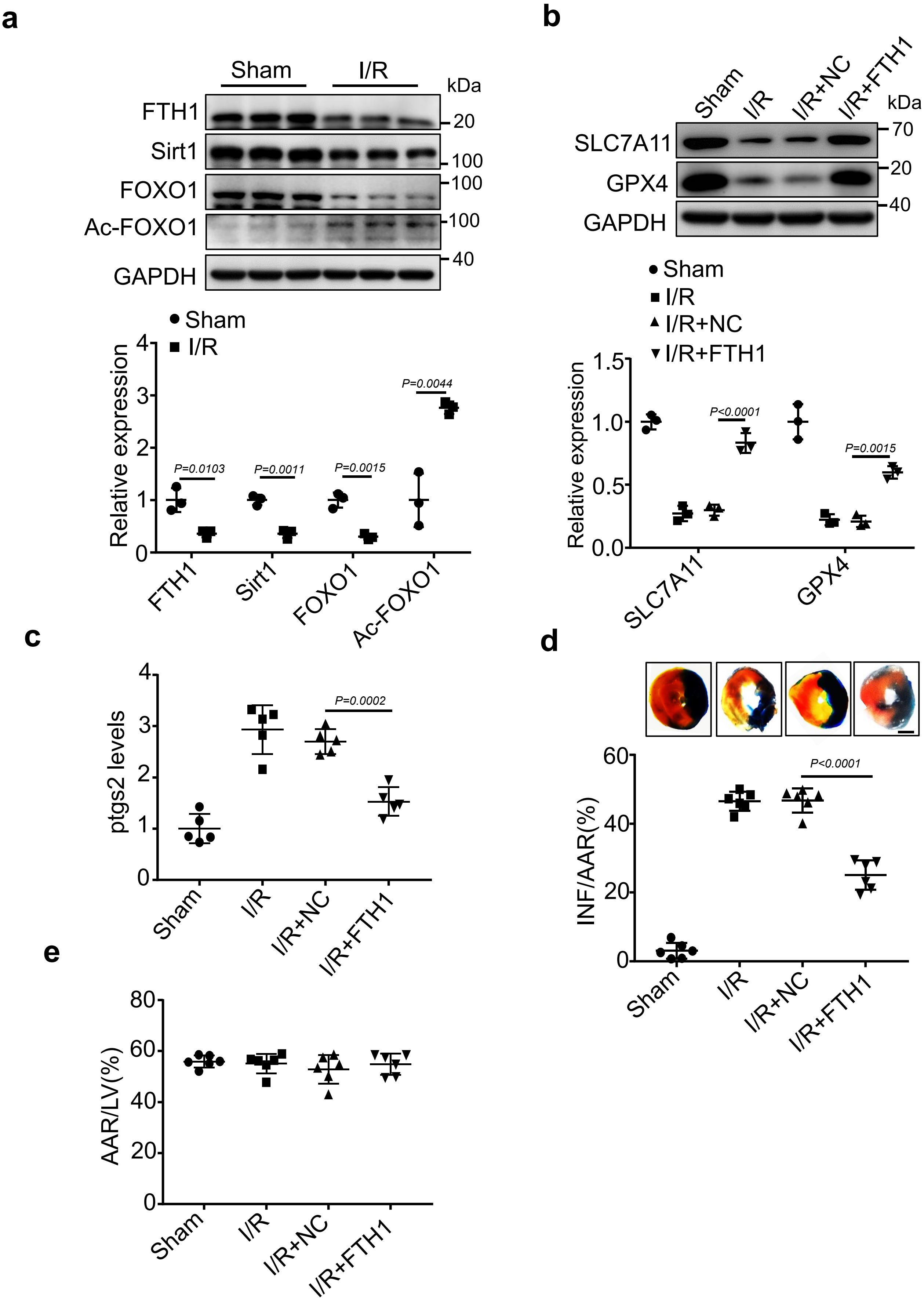

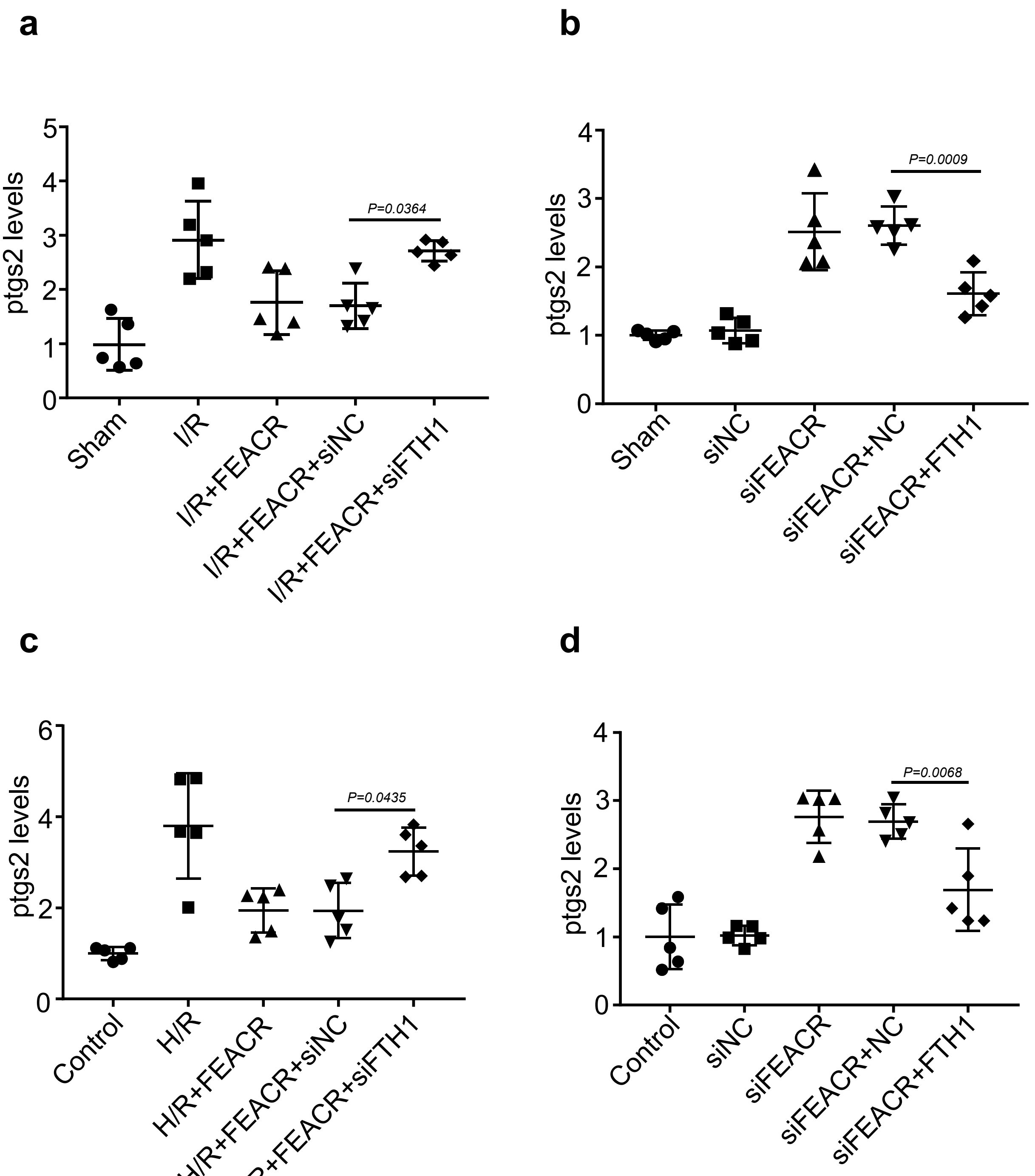
a
ptgs2 levels
0
1
2
3
4
5
P=0.0364
Sham
I/R
I/R+FEACR
I/R+FEACR+siNC
I/R+FEACR+siFTH1
b
ptgs2 levels
0
1
2
3
4
P=0.0009
Sham
siNC
siFEACR
siFEACR+NC
siFEACR+FTH1
c
ptgs2 levels
0
2
4
6
P=0.0435
Control
H/R
H/R+FEACR
H/R+FEACR+siNC
H/R+FEACR+siFTH1
d
ptgs2 levels
0
1
2
3
4
P=0.0068
Control
siNC
siFEACR
siFEACR+NC
siFEACR+FTH1

Supplementary Figure 13

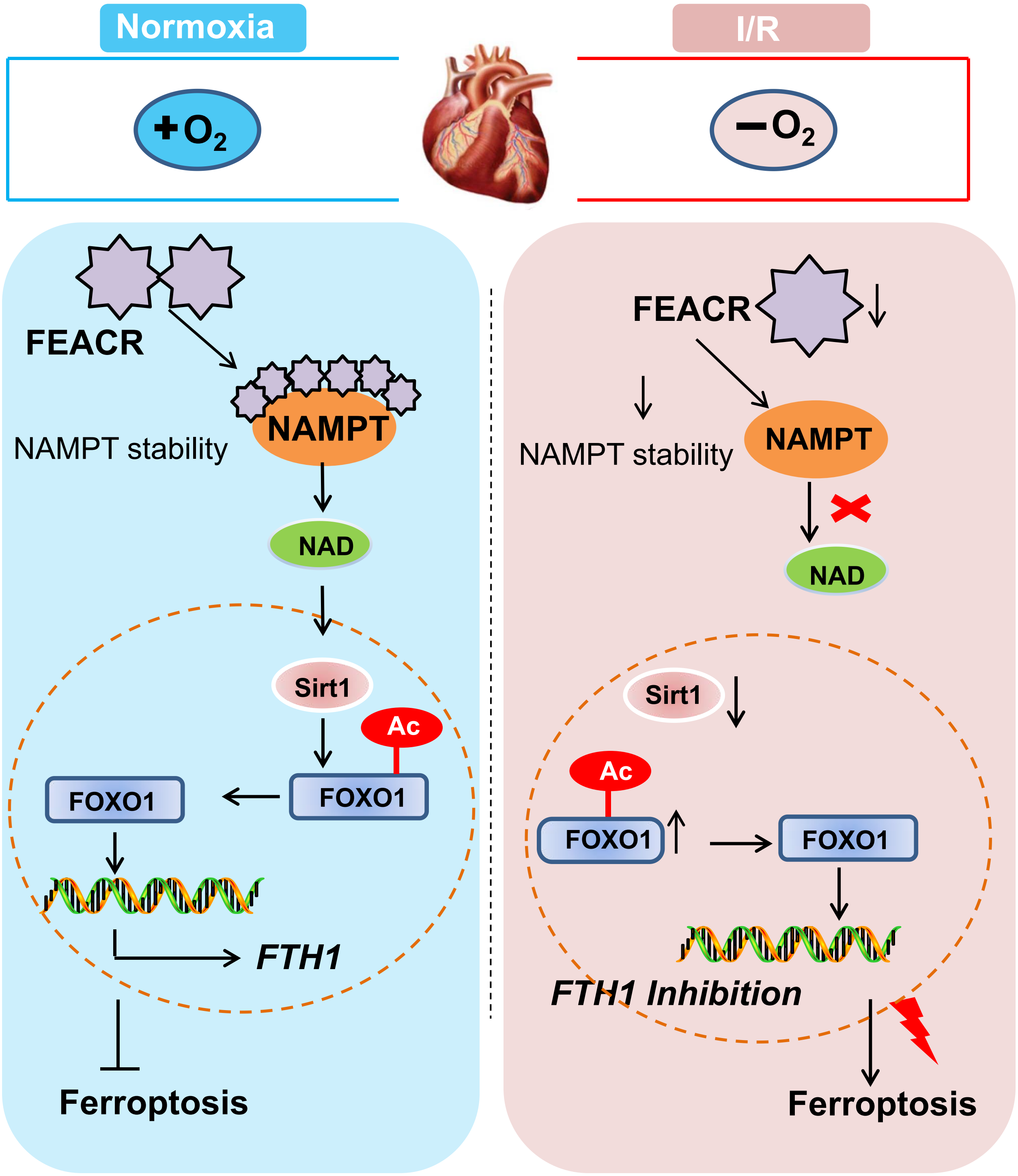

Supplement: Supplementary file 1 — Additional file 1: Supplemental figures and methods. [file 12929_2023_927_MOESM1_ESM.pdf]
